# Heterogeneously Catalyzed Aerobic Oxidation of Methane to a Methyl Derivative

**DOI:** 10.1002/anie.202104153

**Published:** 2021-07-05

**Authors:** Andrea N. Blankenship, Manoj Ravi, Mark A. Newton, Jeroen A. van Bokhoven

**Affiliations:** ^1^ Institute for Chemical and Bioengineering ETH Zurich Vladimir-Prelog-Weg 1 8093 Zurich Switzerland; ^2^ Laboratory for Catalysis and Sustainable Chemistry Paul Scherrer Institute 5232 Villigen Switzerland

**Keywords:** cobalt, fluorous solvents, heterogeneous catalysis, methyl ester, product protection

## Abstract

A promising strategy to break through the selectivity‐conversion limit of direct methane conversion to achieve high yields is the protection of methanol via esterification to a more stable methyl ester. We present an aerobic methane‐to‐methyl‐ester approach that utilizes a highly dispersed, cobalt‐containing solid catalyst, along with significantly more favorable reaction conditions compared to existing homogeneously‐catalyzed approaches (e.g. diluted acid, O_2_ oxidant, moderate temperature and pressure). The trifluoroacetic acid medium is diluted (<25 wt %) with an inert fluorous co‐solvent that can be recovered after the separation of the methyl trifluoroacetate via liquid–liquid extraction at ambient conditions. Silica‐supported cobalt catalysts are highly active in this system, with competitive yields and turnovers in comparison to known aerobic transition metal‐based catalytic systems.

## Graphical abstract

Methane is aerobically converted to an oxidation‐resistant methyl ester in high yields with a solid cobalt‐containing catalyst at a moderate temperature and pressure. Improved reaction conditions result from the dilution of the trifluoroacetic acid in an inert perfluoroalkane co‐solvent. The fluorous character of the solvent enables the facile recovery of the product ester through a simple liquid‐liquid extraction with non‐fluorous solvent.

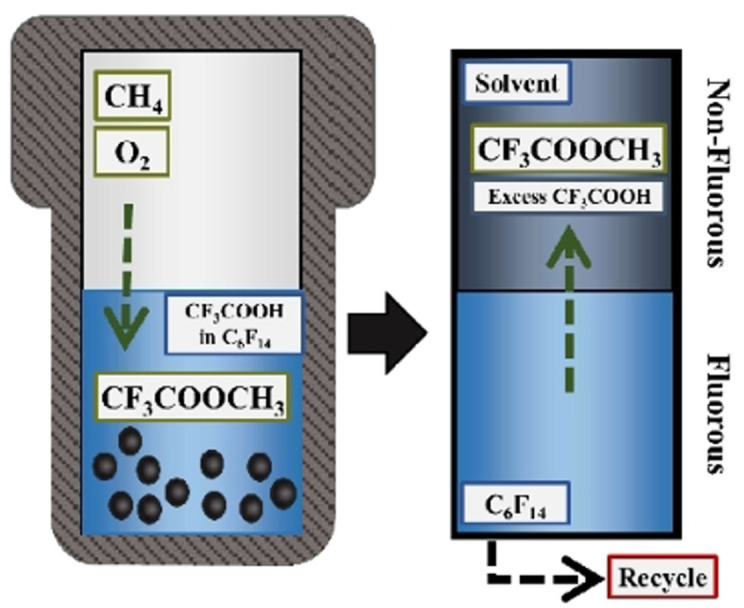

## Introduction

In 2018, it is estimated that nearly 145 billion cubic meters of unused natural gas, a methane‐rich by‐product of oil extraction, was flared owing to the lack of commercial technologies and incentives to bring it to market.^[1]^ Methane derived from natural gas is a highly abundant resource that is widely used in the production of commodity chemicals and liquid energy carriers, notably methanol.^[2]^ Although well‐established industrial routes for methane utilization exist, these processes generally proceed through an indirect, two‐step pathway, with syngas as an intermediate product. Consequently, these energy‐ and capital‐intensive processes are rendered economically unviable for methane valorization at small‐ and mid‐scale facilities,[^2a^, ^3^] such as remote and decentralized shale oil production sites.^[4]^ The challenge of developing a more scale‐flexible direct methane conversion route has therefore motivated both the academic and industrial communities over the previous years.^[5]^

Many approaches have evolved to address this challenge, including the selective functionalization of the methane C−H bond to form primary oxygenates, such as methanol or other derivatives, over heterogeneous catalysts.[^2b^, ^4c^, ^5d^, ^6^] However, existing approaches to partial oxidation are constrained by a number of limitations, most notably, the selectivity‐conversion paradigm arising from the vulnerability of methanol to over‐oxidation.[^5d^, ^7^] Comparisons between diverse solid catalyst systems reveal a clear trend of decreasing selectivity to methanol with increasing conversion of methane, a limit that severely restricts achievable yields and necessitates operating conditions that are highly impractical for commercialization.[^5d^, ^7b^, ^7c^, ^8^] In order to achieve industrially relevant methane conversion without compromising high selectivity for methanol, it is imperative that methanol is stabilized to prevent over‐oxidation.[^7b^, ^7c^, ^9^] An interesting “product protection” strategy, demonstrated primarily in homogeneous catalytic systems, is the protection of methanol via esterification to methyl bisulfate^[10]^ and methyl trifluoroacetate.[^11^, ^18^, ^23^] These esters are less prone to further oxidation under typical reaction conditions^[7b]^ and, therefore, offer the possibility to circumvent the selectivity‐conversion limit during methane conversion. In later steps, these esters can be hydrolyzed back to methanol, thereby widening the array of potential commercial applications for this chemistry. The translation of this protection strategy to use with a heterogeneous catalyst has been largely unexplored, with the notable exception of Schüth and co‐workers who synthesized a highly active solid catalyst based on the homogeneous Periana platinum bipyrimidine complex for the conversion of methane to methyl bisulfate in oleum using a SO_3_ oxidant.^[6e]^

The attractive yields of current methane‐to‐methyl ester processes notwithstanding, these processes still fall short of potential commercial application. Rather than focusing only on obtaining high product yields, a more holistic consideration of process conditions, cost and handling of reagents, and necessary separations and recycle streams is essential. These factors, which have been rigorously defined by industrial experts in this field,^[12]^ constitute a further set of criteria for commercialization. In particular, several key factors have precluded commercialization of these processes, which include: 1) the homogeneous nature of the catalysis, translating into challenges in product and catalyst recovery; 2) the use of strong corrosive acids in an undiluted form, which leads to greater operational hazards and costly equipment;^[13]^ 3) the use of economically unacceptable oxidants, such as potassium persulfate and hydrogen peroxide;^[13]^ and 4) the difficulty in hydrolyzing the ester to methanol due to the highly exothermic interaction of water with the reaction mixture, which potentially results in the undesired evaporation of methanol.^[14]^ A process that demonstrates the breakthrough performance of methane‐to‐methyl‐ester systems while simultaneously overcoming these conventional challenges has not, to our knowledge, been previously demonstrated.

To address the pressing limitations of syngas‐free methane conversion, we propose an approach that combines the heterogeneously catalyzed partial oxidation of methane with subsequent esterification of the product in a reaction medium of trifluoroacetic acid (TFA) diluted in an inert perfluoroalkane co‐solvent. Perfluoroalkanes, such as perfluorohexane, are inert, stable at elevated temperatures,^[15]^ and can exhibit high solubilities for gases.^[16]^ By diluting TFA to manageable concentrations of below 25 wt % in a non‐corrosive and oxidation‐resistant perfluoroalkane, a number of improvements are attained, namely: a strongly reduced corrosivity of the reaction medium; improved stability of a heterogeneous catalyst through operation in a milder environment; enhanced recovery of the methyl ester via a simple liquid‐liquid extraction with a non‐fluorous polar solvent; and improved hydrolysis conditions.

## Results and Discussion

**Figure 1:**
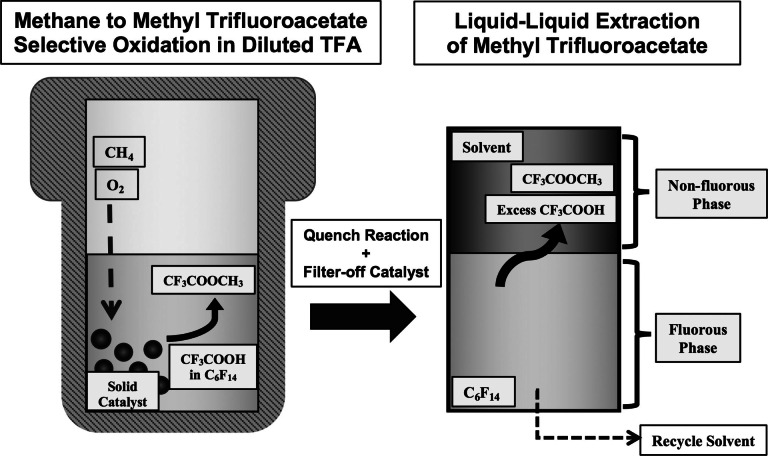
Overview of designed methane oxidation process and product recovery. The conversion of methane to methyl trifluoroacetate occurs in an autoclave charged with methane, air, solid catalyst, and a solution of TFA in perfluorohexane. After reaction, methyl trifluoroacetate is extracted from the fluorous reaction medium into a non‐fluorous solvent.

Previous studies have shown that a number of homogeneous transition metal‐based catalysts display activity for methane to methyl trifluoroacetate conversion, including those based on copper,^[11c]^ manganese,^[11b]^ and cobalt.^[23]^ As a preliminary step, therefore, a variety of transition metals on solid supports were synthesized and screened for activity in a batch reactor system (Figure 1). Based on the initial screening results presented in the Supporting Information, cobalt‐containing silica catalysts synthesized via an incipient wetness impregnation (Co/SiO_2_‐IWI) with an aqueous cobalt nitrate solution showed the most promising activity.

The methyl ester is recovered from the fluorous reaction medium through a facile liquid–liquid extraction with a polar solvent at room temperature. Acetonitrile‐d3 is an aprotic polar solvent used as the extractant to preserve the product as the methyl ester and directly measure the product concentrations using ^1^H NMR. Only residual amounts of the methyl ester remain in the fluorous phase immediately after contact with the deuterated acetonitrile phase as determined through ^1^H NMR (Supporting Information, Figures S1 and S2), and therefore the total product yield can be determined from the deuterated acetonitrile phase after the extraction. Substituting water as the non‐fluorous extracting solvent could provide opportunities for product separation with enhanced ester hydrolysis conditions compared to processes that rely on undiluted reaction mediums. Importantly, this simple and highly effective product separation method provides a straightforward pathway for recycling the fluorous co‐solvent, thereby greatly reducing the overall usage of this component. This unique advantage in product separation and solvent recycle has not been previously described in published methane‐to‐methyl‐ester systems.

**Figure 2:**
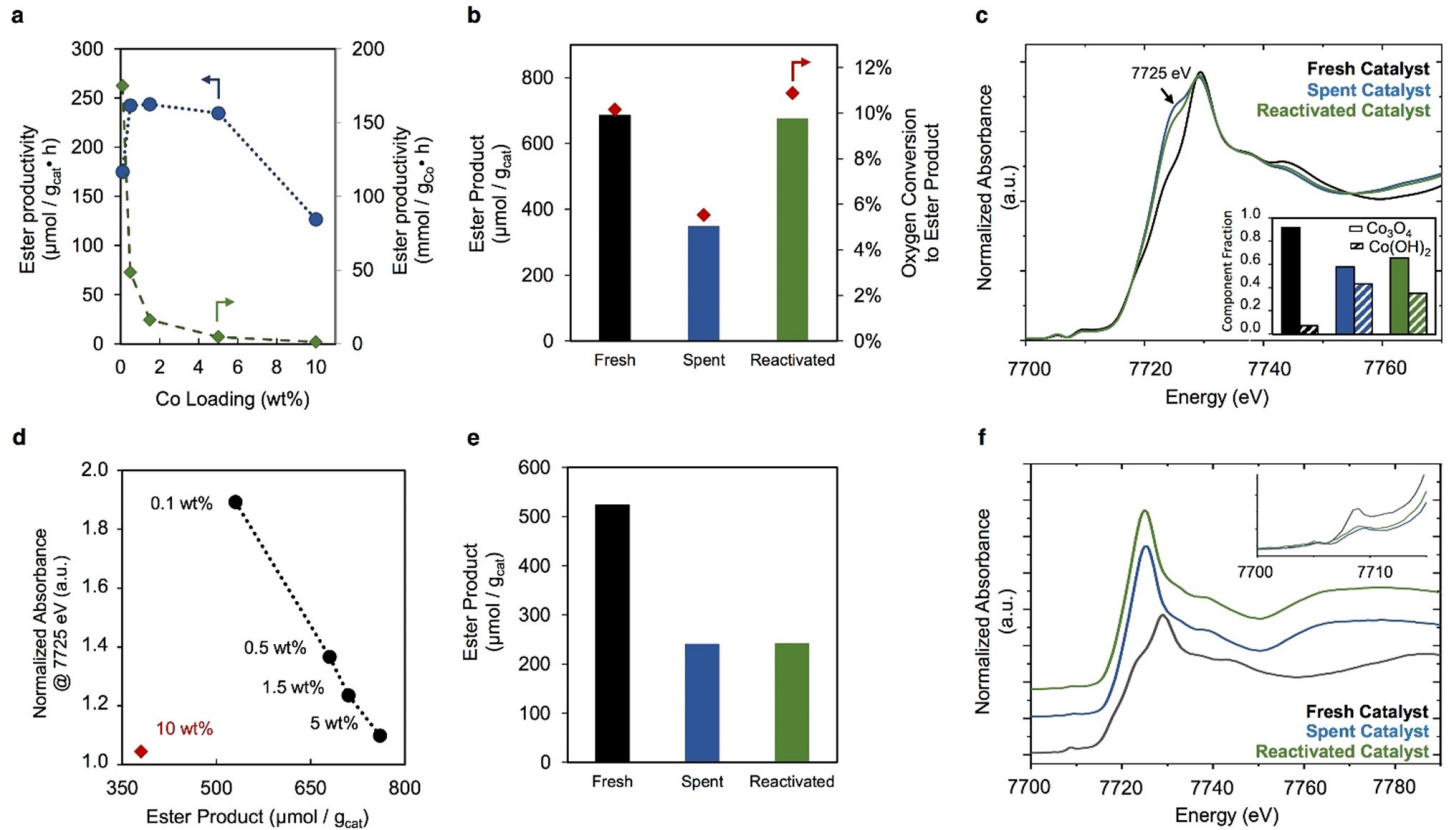
Co/SiO_2_‐IWI catalytic methane oxidation performance and characterization. a) Effect of cobalt loading of Co/SiO_2_‐IWI catalyst on methyl ester productivity in methane oxidation. Reaction conditions: 100 mg catalyst, 5 bar CH_4_, 2 bar air, 7 g of 14 wt % TFA/C_6_F_14_, 215 °C, 3 h; b) Methyl ester product (bar graph) and ester yield (scatter point) obtained with fresh, spent and reactivated 0.5 wt % Co/SiO_2_‐IWI catalyst. Reaction conditions: 5 bar CH_4_, 2 bar air, 7 g of 14 wt % TFA/C_6_F_14_, 215 °C, 100 mg catalyst, 3 h; c) Co K‐edge XANES of 0.5 wt % Co/SiO_2_‐IWI series. Inset includes two‐component LCA results with the Co_3_O_4_ and Co(OH)_2_ standards; d) Normalized absorbance of the Co K‐edge XANES at 7725 eV for the spent Co/SiO_2_‐IWI catalysts with respect to corresponding ester product of the catalyst during reaction; e) Methyl ester product obtained with fresh, spent and reactivated 0.1 wt % Co/SiO_2_‐IWI catalyst. Reaction conditions: 5 bar CH_4_, 2 bar air, 7 g of 14 wt % TFA/C_6_F_14_, 215 °C, 100 mg catalyst, 3 h; f) Co K‐edge XANES of 0.1 wt % Co/SiO_2_‐IWI series. Inset includes close‐up of pre‐edge feature.

Herein, catalysts with cobalt loadings of 0.1 wt %, 0.5 wt %, 1.5 wt %, 5 wt %, and 10 wt % were synthesized via an incipient wetness impregnation method (Co/SiO_2_‐IWI) and studied for the catalytic partial oxidation of methane. Figure 2 a illustrates the dependence of the methyl ester productivity on the cobalt loading of the Co/SiO_2_‐IWI catalysts under the typical reaction conditions. A methyl ester productivity of approximately 250 μmol g_cat_^−1^ h^−1^ is obtained with the 0.5 wt % Co/SiO_2_‐IWI catalysts. Increasing the cobalt content of the catalysts up to 5 wt % results in only minor changes in the productivity of the methyl ester (Figure 2 a, blue markers). Cobalt utilization is substantially higher for the low‐loaded catalysts, with a maximum of nearly 175 mmol g_Co_^−1^ h^−1^ achieved using the 0.1 wt % Co/SiO_2_‐IWI material, which then decreases rapidly with increasing cobalt loading (Figure 2 a, green markers). The performance of the 10 wt % Co/SiO_2_‐IWI catalyst is substantially inferior to the catalysts with lower cobalt content in terms of both productivity and cobalt utilization. In combination with the average particle sizes from the corresponding transmission electron microscopy (TEM) images (see Supporting Information), these results suggest that the more active catalysts are those with the higher cobalt dispersions. Consequently, the most efficient utilization of cobalt is realized in the lowest cobalt‐loaded cases.

The heterogeneity of the reaction for the Co/SiO_2_‐IWI materials was confirmed via a hot filtration test and ICP‐OES of the reaction medium (Supporting Information, Figure S4). Additional tests demonstrating the necessity of cobalt and molecular oxygen oxidant are summarized in Table S3 of the Supporting Information. To further assess the stability of the Co/SiO_2_‐IWI catalysts, the catalyst material was recovered after an initial catalytic test and recycled back into the reactor for a second run conducted under the same conditions. Before recycling, some samples underwent a thermal treatment consisting of a calcination to at least 250 °C in static air and are denoted as “reactivated” samples. Samples that did not receive any treatment before the second run are referred to as “spent” samples. The catalytic productivity obtained with the spent 0.5 wt % Co/SiO_2_‐IWI catalyst after reactivation is comparable to that obtained with the freshly synthesized catalyst (Figure 2 b). Without the reactivation step, this catalyst demonstrates decreased activity.

On inspecting the fresh catalysts by TEM, we find large cobalt‐containing particles in those with high loadings, and X‐ray diffraction (XRD) of these catalysts confirms the presence of Co_3_O_4_ (see Supporting Information). X‐ray absorption spectroscopy (XAS) further corroborates the dominance of a spinel Co_3_O_4_ at each of the weight loadings in the fresh state and suggests the presence of a minority Co^II^‐like fraction, which becomes less significant as the cobalt loading increases (Supporting Information, Figure S9). Figure 2 c shows the Co K‐edge X‐ray absorption near edge structure (XANES) of the 0.5 wt % Co/SiO_2_‐IWI catalyst in the fresh, spent, and reactivated state. The lower binding energy feature around 7725 eV associated with a Co^II^ component is noticeably more prominent in the spent and reactivated catalysts as compared to the fresh catalysts. Linear combination analysis (LCA) fits to the XANES of these materials (see Supporting Information) suggests that the reactivated catalyst regains more of the Co^III^/Co^II^ character indicative of the initial spinel structure, but still retains a significant, loading‐dependent amount of the second Co(OH)_2_‐like character (Figure 2 c, inset). For the higher‐loaded samples, such as the 5 wt % Co/SiO_2_‐IWI catalyst, the cobalt structure appears to be less perturbed by reaction in the spent catalysts and more closely resembles the initial structure following reactivation (Supporting Information, Figure S11).

In the case of the 0.1 wt % Co/SiO_2_‐IWI catalysts, both the spent and reactivated catalysts show equivalent ester productivities that are approximately 50 % of that obtained with the fresh catalyst (Figure 2 e). Despite the decrease in activity observed after the initial reaction, the spent and reactivated samples still show better cobalt utilization than the higher‐loaded catalysts, with cobalt‐based ester productivities around 80 mmol g_Co_^−1^ h^−1^ (corresponding to approx. 240 μmol g_cat_^−1^ in Figure 2 e). From an activity standpoint, the aerobic reactivation protocol does not have a significant effect on the 0.1 wt % catalyst. The XAS of both the spent and reactivated materials that reveals a strong resemblance between these materials (Figure 2 f) supports this observation. The effects of catalysis manifest principally through the development of a shoulder in the XANES, associable with Co^II^ @ 7725 eV in the higher‐loaded samples (Figure 2 c). There is an explicit linear relationship between the fraction of cobalt associated with the 7725 eV Co^II^ XANES feature and the obtained methyl ester yield over the studied range of cobalt loadings (Figure 2 d). The 10 wt % Co/SiO_2_‐IWI catalyst noticeably deviates from this trend, which likely results from the greatly increased domain size and reduced dispersion due to the formation of very large cobalt particles and agglomerates, as evidenced by TEM and XRD (see Supporting Information). In the case of the 0.1 wt % material, the XANES feature @7725 eV Co^II^ is transformed into a strong peak rather than a shoulder (Figure 2 f), and the overall XANES envelopes of 0.1 wt % Co/SiO_2_‐IWI in the spent and reactivated state are markedly different from those of the higher weight‐loaded samples.

The pre‐edge feature at ca. 7709 eV is indicative of a fraction of the cobalt that initially exists with the tetrahedral (T_d_) symmetry expected from the Co^II^ component of the spinel structure of Co_3_O_4_ (Figure 2 f, inset).^[17]^ This feature is removed in the spent catalyst, suggesting the removal of T_d_ symmetry from the 0.1 wt % sample post‐reaction. Moreover, the overall shift of the Co K‐edge edge position to lower energy further suggests that the octahedral (O_h_) Co^III^ component of the Co_3_O_4_, has also been consumed. Analysis of the EXAFS further indicates that a complete structural transformation of the cobalt species present on the 0.1 wt % catalyst from the Co_3_O_4_ starting phase into a highly dispersed O_h_ Co^II^ species has occurred (see Supporting Information). The combination of catalytic data with the XAS suggests that the new O_h_ Co^II^ species can still be associated with activity for methane oxidation. The most active cobalt species across the series of catalysts, therefore, may not be the Co_3_O_4_ nanoparticles that are predominantly present in the fresh catalysts across the range of loadings, but rather a newly formed species that is best observed in the low‐loaded, highly dispersed catalyst materials.

**Figure 3:**
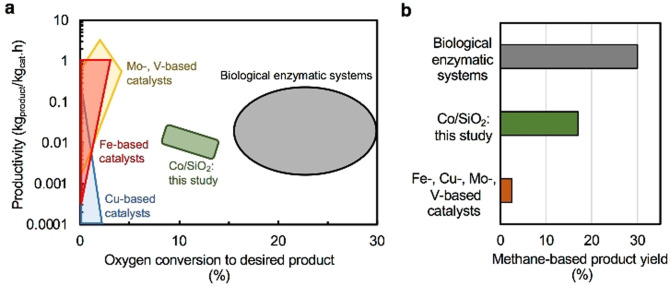
Overview of the performance of different aerobic methane oxidation approaches. a) Comparison of the productivity and oxygen‐based yields of previously reported methane‐oxidation with molecular oxygen approaches; b) Comparison of the methane‐based product yields of various methane‐oxidation with molecular oxygen approaches.

By increasing the liquid hold‐up in the reactor and reducing the partial pressure of oxygen, the highest oxygen‐based yield of 12 % of the theoretical maximum was reached with 1.5 wt % Co/SiO_2_ catalyst (Supporting Information, Table S5). Figure 3 a ranks the performance of the cobalt‐catalyzed process reported herein in comparison to other methane oxidation approaches that solely use dioxygen as the oxidant based on two important parameters: productivity and oxygen conversion to the desired product. This comparison reveals the considerable performance‐gap that exists between aerobic heterogeneous systems for methane‐oxidation. The latter operate aerobically with a NADH electron carrier and are characterized by a highly selective conversion of methane to methanol.^[26]^

As high‐temperature heterogeneous catalytic approaches using different transition metals typically result in a much poorer selectivity even at lower conversion, these systems appear on the very left in Figure 3 a. The performance of the Co/SiO_2_‐IWI catalyst is distinct from these other approaches and advances toward the more efficient bio‐enzymatic systems. Furthermore, methanol productivity achieved per gram of the cobalt catalyst is of the same order as that achieved per gram of dry/ wet cells in the biological conversion of methane. With the Co/SiO_2_‐IWI catalyst, a productivity up to 0.03 kg_methanol_ kg_cat_^−1^ h^−1^ was attained at low cobalt loadings under low‐pressure conditions and a pronounced enhancement is expected on increasing the feed pressure further. The volumetric productivity, or space‐time‐yield (STY), is an additional metric useful for comparing processes. Although optimization of this parameter was not a focus of this work, volumetric productivity in this case would likely be greatly enhanced through increasing the amount of catalyst in the reactor, increasing methane partial pressures, and targeting the retention of efficient cobalt utilization at higher weight loadings.

Apart from excellent oxygen‐based product yields, working at lower partial pressure of methane showed improved selective methane conversion to the methyl ester. Reactions performed with a 2.5 % methane feed resulted in methane‐based ester yields of up to 17 % (Supporting Information, Table S4). While the transition metal‐based catalysts depicted in Figure 3 b reach 2–3 % methane‐based yields at best, biological systems consistently report yields close to 30 %.^[26]^ The high activity of MMO enzymes coupled with the high selectivity in methane conversion and oxygen utilization has been proven difficult to be replicated in heterogeneous systems. The high activity of the cobalt‐based catalyst along with the use of esterification as a “product protection” strategy enables a performance that is comparable to the bio‐enzymatic systems.

Table 1 lists the activity of the Co/SiO_2_‐IWI catalyst with known homogeneous catalytic systems that oxidize methane to methyl trifluoroacetate. The performance of these systems is compared on the basis of the reported turnovers (TO) over the reaction period and the productivity. We note that the turnovers reported typically represent a lower bound on the amount of product formed per mole of catalyst, since in most cases it appears that the catalyst may not be fully deactivated at the end of the reaction period. The Co/SiO_2_‐IWI catalyst achieved turnovers of up to 31 and a corresponding productivity of 10.3 mol_ester_ mol_Co_^−1^ h^−1^. This performance is competitive to even homogeneous systems that employ stronger oxidants (K_2_S_2_O_8_, H_2_O_2_), higher partial pressures of methane, and/or more complex redox cycling schemes. Taken together, Figure 3 and Table 1 illustrate the outstanding catalytic performance of the system that is achieved with an economical oxidant and under reaction conditions that are more favorable than those generally employed in state‐of‐the‐art methane‐to‐methyl‐ester systems.

**Table 1:** A comparison to homogeneous systems for the oxidation of methane to methyl trifluoroacetate.

Entry	Pre‐catalyst	Oxidant	P_methane_ [bar]	Reported TO^[a]^	Productivity^[b]^ [mol_ester_ mol_metal_^−1^ h^−1^]	Ref.
1	Pd(OAc)_2_	K_2_S_2_O_8_	20	3.8	0.2	[18]
2	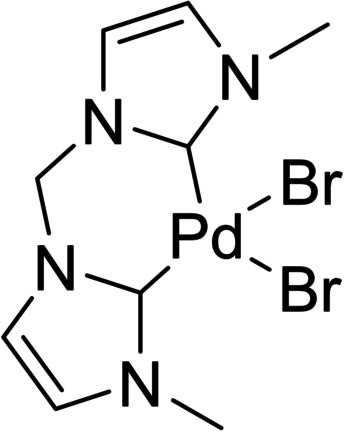	K_2_S_2_O_8_	30	30	2.1	[18]
3	H_5_PV_2_Mo_10_O_40_	K_2_S_2_O_8_	10	128^[c]^	6.4^[c]^	[19]
4	CuO	K_2_S_2_O_8_	5	33	1.9	[11c]
5	Co(OCOCF_3_)_3_	O_2_	20	4	1	[20]
6	EuCl_3_/Zn	O_2_	10	5.3	5.3	[21]
7	FeCl_3_	O_2_	10	<0.5	<0.5	[21]
8	Pd(OAc)_2_/BQ/H_5_PMo_10_V_2_O_40_	O_2_	25	3–118^[d]^	0.4–14.8^[d]^	[22]
9	Co(OAc)_2_.4H_2_O	O_2_	20	13.2	0.6	[23]
10	Mn_2_O_3_	O_2_	7	8.5	2.8	[11b]
11	[Pd(hfacac)_2_]	H_2_O_2_	30	39	9.8	[24]
12	[Cu(hfacac)_2_(H_2_O)_2_]	H_2_O_2_	30	13	3.3	[24]
13	VO(acac)_2_	H_2_O_2_	50	18.5^[c]^	0.8^[c]^	[25]
14	H_4_PV_1_Mo_11_O_40_	H_2_O_2_	50	224^[c]^	9.3^[c]^	[25]
15	0.1 % Co/SiO_2_‐IWI	O_2_	5	31	10.3	This study

[a] [Moles of methyl trifluoroacetate]/ [Moles of metal in catalyst] measured over the reported reaction time; [b] Average rate of production based on reported metal/catalyst loading and reaction time; [c] Including methyl acetate as a product; [d] Determined with respect to Pd(OAc)_2_.

## Conclusion

An improved methane‐to‐methyl ester process with competitive performance for the selective conversion of methane was designed by systematically addressing the limitations of conventional systems. The use of a fluorous co‐solvent as an acid diluent brought several advantages, notably a milder reaction environment that allowed working in a heterogeneous mode and a facile product and solvent recovery method through a highly effective extraction with a non‐fluorous solvent. The performance of this cobalt‐catalyzed process far exceeds other comparable heterogeneous transition metal‐based high‐temperature catalytic processes, which are generally restricted to much lower methane conversion and product selectivity. Through future work to elucidate the reaction mechanisms, target improvement of the cobalt utilization at high metal loadings, and optimization of process conditions, this novel catalytic approach holds the promise of significant future improvement.

## Conflict of interest

The authors declare no conflict of interest.

## Supporting information

As a service to our authors and readers, this journal provides supporting information supplied by the authors. Such materials are peer reviewed and may be re‐organized for online delivery, but are not copy‐edited or typeset. Technical support issues arising from supporting information (other than missing files) should be addressed to the authors.

Supporting Information
