# Supplementary material for: Heterogeneously Catalyzed Aerobic Oxidation of Methane to a Methyl Derivative
Source: Angew Chem Int Ed Engl. 2021 Jul 5;60(33):18138–43. doi: 10.1002/anie.202104153 (PMC8456920; doi:10.1002/anie.202104153)
Supplement: Supplementary file 1 — Supporting Information [file ANIE-60-18138-s001.pdf]

Angewandte Chemie
Eine Zeitschrift der Gesellschaft Deutscher Chemiker

Supporting Information

Heterogeneously Catalyzed Aerobic Oxidation of Methane to a Methyl Derivative

*Andrea N. Blankenship[+], Manoj Ravi[+], Mark A. Newton, and Jeroen A. van Bokhoven**

anie_202104153_sm_miscellaneous_information.pdf

TABLE OF CONTENTS

Methods 3
Synthesis of Incipient Wetness Impregnation (IWI) Catalysts 3
Synthesis of Liquid-phase Deposition (LPD) Catalysts 3
Conversion of methane to methyl trifluoroacetate 3
Hot Filtration Test 3
Product Analysis 4
Catalyst Characterization 4
Safety – Flammability Limits of Methane/Air Mixtures 5
Recovery of Methyl Trifluoroacetate 7
Catalyst Screening 9
Co/SiO_2-LPD Catalytic Activity and Characterization 10
Co/SiO_2-IWI Catalytic Activity 12
Co/SiO_2-IWI Characterization of Catalysts 14
TEM Images and Particle Size Distribution 14
XRD 16
Co K-Edge XANES in Fresh State 17
LCA Analysis of the Co K-Edge XANES 18
Co K-Edge EXAFS in Fresh State 21
Co K-Edge XANES and EXAFS in Spent State 22
EXAFS Fitting of the Spent 0.1 wt% Co/SiO_2-IWI Catalyst 23
Fluorous Co-Solvent Selection and Effects 27
Ester Hydrolysis 29

LIST OF SUPPORTING FIGURES

Fig. S1. ^{1}H NMR Spectra Of The Two Phases After LLE Extraction – Perfluorohexane 7
Fig. S2. ^{1}H NMR spectra of the two phases after LLE extraction – Perfluorooctane 8
Fig. S3. Co/SiO_2-LPD catalytic methane oxidation performance and characterization 11
Fig. S4. Hot filtration test for Co/SiO_2-IWI 12
Fig. S5. TEM images of Co/SiO_2-IWI catalysts 14
Fig. S6. EDXS representative of Co/SiO_2-IWI catalyst with 0.5 wt% Co loading 15
Fig. S7. EDXS representative of Co/SiO_2-IWI catalyst with 1.5 wt% Co loading 15
Fig. S8. XRD of Co/SiO_2-IWI catalysts in fresh state 16
Fig. S9. Co K-edge XANES of Co_3O_4, CoO, and $Co(OH)_2$ standards and Co/SiO_2-IWI catalysts in fresh state 17
Fig. S10. Variation of X^2 resulting from three different approaches to a LCA fitting of the Co K-edge XANES for the Co/SiO_2-IWI catalysts in their fresh, spent, and reactivated states 19
Fig. S11. Comparison of the fits to Co K-edge XANES achieved from the two-component LCA (Co_3O_4 + $Co(OH)_2$) for the 0.1 wt% and 5 wt% Co/SiO_2-IWI catalysts in the fresh, spent, and regenerated states 20
Fig. S12. EXAFS of all Co/SiO_2-IWI catalysts in fresh state 21
Fig. S13. XAS summary of all Co/SiO_2-IWI catalysts in spent state 22
Fig. S14. Initial fitting of the Co K-edge EXAFS derived from the 0.1 wt% Co/SiO_2-IWI sample using full cured wave multiple scattering theory for two candidate symmetries as indicated. 23
Fig. S15. EXAFS analysis for 0.1 wt% Co/SiO_2-IWI in spent state 24
Fig. S16. Effect of nature of perfluorocarbon co-solvent on activity of 1.5 wt% Co/SiO_2-IWI catalyst 28
Fig. S17. TEM of Co/SiO_2-IWI catalyst in spent state 28

LIST OF SUPPORTING TABLES

Table S1. Catalytic activity screening of various transition metal-based catalysts 9
Table S2. Catalytic and aerobic conversion of methane to methyl ester for Co/SiO_2-LPD catalyst 11
Table S3. Catalytic and aerobic conversion of methane to methyl trifluoroacetate for Co/SiO_2-IWI catalysts 12
Table S4. Methane-based yields for Co/SiO_2-IWI 1.5 wt% in oxygen-rich conditions 13
Table S5. Oxygen-based yields for Co/SiO_2-IWI 1.5 wt% in methane-rich conditions 13
Table S6. Particle size distribution of Co/SiO_2-IWI catalysts based on TEM images 14
Table S7. Structural and statistical data arising from analysis of Co K-edge EXAFS from the spent 0.1 wt% Co/SiO_2-IWI catalyst for the six models shown in Figure S14 26

Methods

Synthesis of Incipient Wetness Impregnation (IWI) Catalysts

An aqueous cobalt nitrate solution was prepared (cobalt (II) nitrate hexahydrate; 99% (ABCR) dissolved in Milli-Q H_2O) and added directly to 1.0 g of silica gel (high purity, 60 Å pore size, 230-400 mesh particle size (Fluka Analytical)) to the desired weight loading (0.1 %, 0.5 %, 1.5 %, 5 %, 10 %). After drying in static air at 80 ºC, the samples were then calcined at 550 ºC for 6 h with a heating rate of 5 ºC/m. The resulting solids ranged from beige to black in colour depending on the weight loading.

Synthesis of Liquid-phase Deposition (LPD) Catalysts

An aqueous solution of a metal salt of the desired transition metal ($Co(NO_3)_2$*$6H_2O$, $Cu(CO_2CH_3)_2$*H_2O, $Mn(CH_3COO)_2$*$4H_2O$) was prepared by dissolving approximately 1.3 mmol of the metal salt into 130 mL deionized H_2O. Next, approximately 7–9 mmol of the desired support material was added to the solution, and the mixture was left to stir for at least 3 h at room temperature. The mixture was then filtered and washed with deionized water. The solids were dried in static air at 80-90 ºC and subsequently calcined at 550ºC for 6 h with a heating rate of 5 ºC/min.

Conversion of methane to methyl trifluoroacetate

Catalyst testing was conducted in a 45 mL Hastelloy autoclave fitted with a 25 mL Teflon insert. The Teflon insert was typically charged with 7 g of 14 wt% trifluoroacetic acid (TFA) in a perfluoroalkane solvent and 100 mg of the catalyst. The Teflon insert was then immediately fitted into the autoclave, and the vessel was subsequently sealed. The vessel was purged at least three times with 5 bar of either CH_4 or air in order to remove any contaminant gases. The vessel was then charged with the desired mixture of gases (e.g. 5 bar CH_4, 2 bar air) and heated to the desired temperature up to a limit of 215 ºC (Note: due to thermal limitations of some components in the set-up, a temperature above 215ºC could not be safely reached.). The vessel was stirred at 500 rpm and the reaction was allowed to proceed for the desired amount of time. To recover the products, the reactor was cooled in ice to quench the reaction. The reactor was cooled below room temperature to reduce the loss of volatile products before collecting a gas sample and opening the vessel to collect the liquid sample.

Hot Filtration Test

A hot filtration test refers to the detection of active species leached from the solid catalyst during reaction by removing the solid catalyst from the liquid reaction medium and testing the filtered reaction medium for activity. After the initial methane oxidation reaction, the catalyst was removed via filtration. Additional fresh (14 wt%) TFA solution was added to the liquid filtrate to make up the original liquid volume. A small sample was taken for NMR analysis to determine the initial ester concentration of the liquid filtrate. The liquid reaction filtrate was then reinserted into the reactor and the reaction continued

under the same procedure as the initial catalytic test. A substantial increase in ester content between the initial and hot filtration tests would therefore reveal the presence of any homogeneous active species in solution. In some instances, ICP-AES was also performed on the reaction filtrate to confirm if leached Co was present homogeneously in the solution.

Product Analysis

The gaseous products were collected in a gas chromatography (GC) gas-sampling bag and analysed by a thermal conductivity detector on Agilent micro GC 3000A fitted with Molsieve (10m long, 0.32mm ID) and PLOTU (8m long, 0.32mm ID) columns after cooling the reactor. Carbon dioxide was detected on the PLOTU column, and then quantified against a calibration curve based on commercial standards. Unreacted methane in the gas phase was detected on the Molsieve column and PLOTU column, but the methane on the PLOTU column could not be clearly resolved from the He carrier gas. Carbon monoxide was not detected in any of the experiments.

The liquid phase products were first extracted by filtering off the solid catalyst and then contacting a sample of the reaction filtrate with deuterated acetonitrile (CD_3CN) solvent under mild agitation. After the visible phase separation between the fluorous and CD_3CN phases, the CD_3CN phase was removed and immediately analysed with ^1H NMR. NMR spectra were recorded at room temperature on a Bruker DPX 300 MHz Ultra-Shield NMR spectrometer. NMR analysis of the remaining fluorous phase after extraction showed no detectable amounts of the methyl ester, therefore quantification of the product was made using only the CD_3CN phase (see Supporting Information Figure S3). The products were quantified against their corresponding calibration curve of peak area versus analyte concentration. After quantification of the products, the ester yield for experiments under oxygen-rich reaction conditions was calculated as:

$$ester\ yield = \frac{mol.of\ ester\ produced}{mol.of\ initial\ methane} * 100$$

The ester yield for experiments under methane-rich conditions was calculated similarly, but using the initial amount of oxygen. The theoretical maximum ester yield was calculated assuming a 100% conversion of the limiting reactant (either molecular oxygen or methane) to the methyl ester. The ester productivity is defined in this study as:

$$ester\ productivity = \frac{mol.of\ ester\ produced}{catalyst\ mass * rxn\ time}$$

Estimating the selectivity was not straightforward due to the presence of CO_2 resulting from the reaction media itself, therefore the discussion of catalytic performance is only reported in terms of the previously defined indicators.

Catalyst Characterization

The catalysts were studied by X-ray diffraction acquired on a PANalytical diffractometer using Cu Kα radiation. Scanning transmission electron microscopy (STEM) investigations of the cobalt-containing

materials were performed on an aberration-corrected, dedicated STEM microscope, a HD-2700CS (Hitachi), operated at an acceleration potential of 200 kV. Samples of the catalyst materials were dispersed in ethanol and a few drops of the suspension were deposited and dried onto a perforated carbon foil supported on a copper grid. The images (1024 × 1024 pixels) were recorded with frame times between 10 and 20 s. Analytical investigations were made with an energy-dispersive X-ray spectrometer (EDXS) attached to the microscope column.

Inductively coupled plasma optical emission spectroscopy (ICP-OES) was used to determine the cobalt content of the synthesized catalysts and the liquid reaction medium using a Horiba Ultra 2 spectrometer. A cobalt calibration curve was prepared from standard solutions. For solid catalyst materials, approx. 10 - 20 mg of the solids was digested in concentrated nitric acid and hydrofluoric acid overnight. The liquid reaction mediums after reaction were tested for leaching by filtering off the solid catalyst, performing the liquid-liquid extraction with Milli-Q H_2O, and then taking a sample from the aqueous phase for analysis.

X-ray absorption spectroscopy (XAS) measurements were made at the Co K-edge at the SuperXAS beamline of the Swiss Light Source using a quick scanning, channel cut Si(111) monochromator[1]. The solid catalysts samples were combined with cellulose, pressed into self-supporting disks using 1 ton of pressure, and sealed in Kapton tape. The relative amounts of cellulose to sample were calculated using XAFSmass software[2]. For samples with cobalt loadings between 0.5 and 10 wt%, the X-ray absorption near edge structure (XANES) and extended X-ray absorption fine structure (EXAFS) at the Co K-edge were acquired both in transmission using fast gridded ion chambers[1] and fluorescence using a PIPS detector[3] in parallel with a spectral acquisition rate of 1 Hz. The 300 QEXAFS scans were then averaged, reduced, and normalized using the ProQEXAFS package[4]. For the 0.1 wt% Co/SiO_2-IWI sample, spectra were collected in the fluorescence mode using a 5 element drifted silicon diode detector. Subsequent analyses of the EXAFS were made using EXCURV[5] and linear combination analyses (LCA) using Prestopronto[6].

Safety – Flammability Limits of Methane/Air Mixtures

The gas compositions used in this study were selected with consideration of the flammability limits of methane/air gas mixtures. Flammability limits are dependent on a multitude of factors, including temperature and pressure[7]. The set-up used in this study involves elevated temperatures and pressure, therefore, consideration of the temperature and pressure dependency of the flammability limits is paramount for safe operation. In particular, the upper flammability limit (UFL) is the most sensitive to increases in temperature and pressure, and results in a broadening of the inoperable range of methane/air compositions. Notably, this can increase this limit well above the 15 vol% methane often cited for methane/air mixtures at room temperature and atmospheric pressure to twice this value near conditions

used in this study[7a]. We choose to operate well above this limit (above 70% methane for the standard reaction conditions), keeping in mind that the composition of the gases inside the reactor can change during reaction. The lower flammability limit (LFL) is lower at increased temperature and pressure, and this was also considered when choosing the oxygen-rich conditions listed in Table S5.

Recovery of Methyl Trifluoroacetate

The methyl ester is recovered from the fluorous reaction medium through a facile liquid-liquid extraction with a polar aprotic solvent, such as acetonitrile, at room temperature. Although TFA and the corresponding methyl ester are miscible in the fluorous co-solvent, upon contact with the polar solvent, they will partition almost entirely out of the fluorous phase. Only negligible amounts of the methyl ester remain in the fluorous phase immediately after contact with the deuterated acetonitrile phase as determined through ^{1}H NMR (Figures S1 and S2), therefore the total product yield can be effectively determined through the analysis of only the acetonitrile phase after extraction.

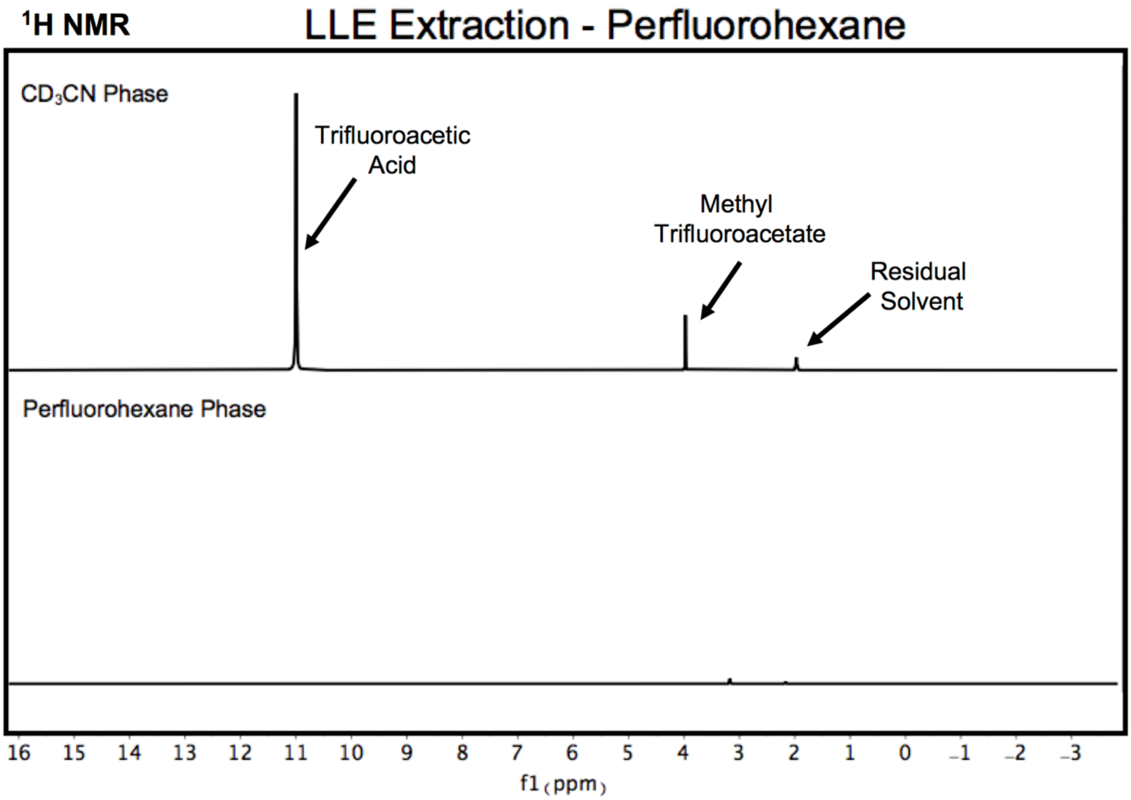

Fig. S1. ^{1}H NMR Spectra Of The Two Phases After LLE Extraction – Perfluorohexane
Taken immediately after the addition of CD3CN to the fluorous phase solution and mild agitation. The perfluorohexane phase shows only residual amounts of the acid and methyl ester after extraction.

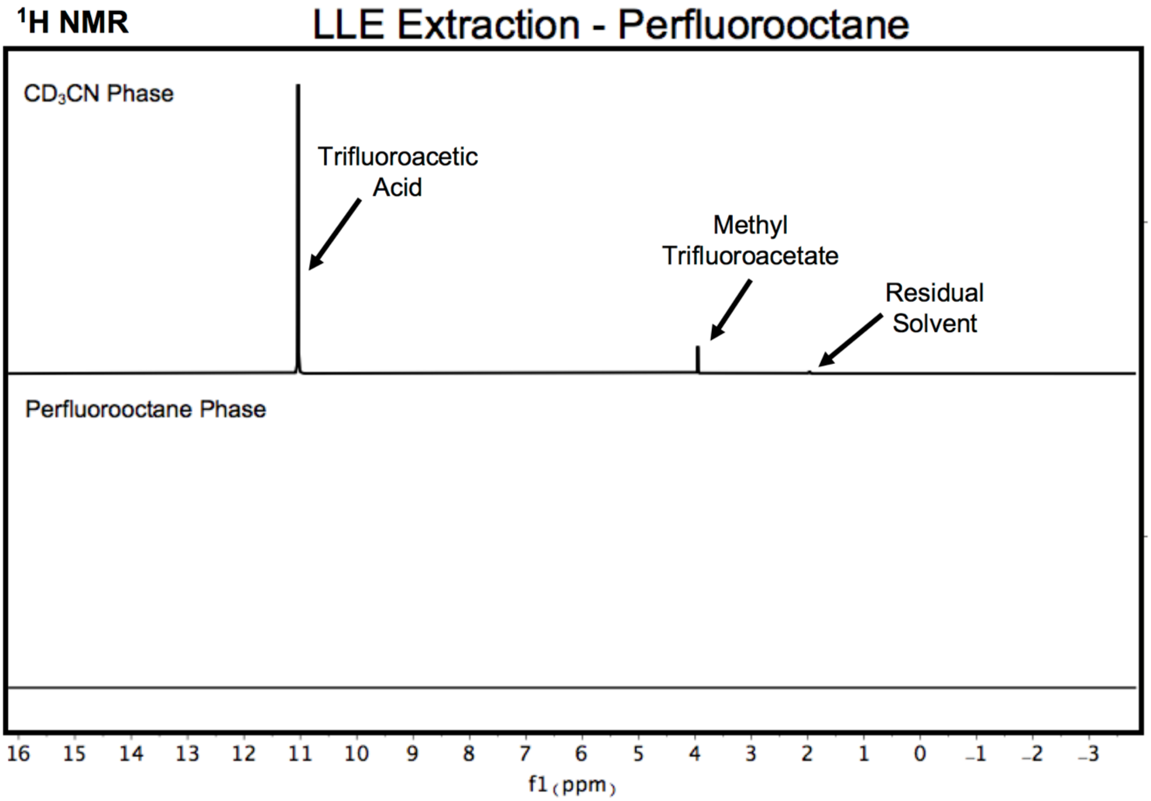

Fig. S2. ¹H NMR spectra of the two phases after LLE extraction – Perfluorooctane
Taken immediately after the addition of CD3CN to the fluorous phase solution and mild agitation. The perfluorooctane phase shows only residual amounts of the acid and methyl ester after extraction.

Catalyst Screening

Previous studies have shown that a number of homogeneous transition metal catalysts show activity for for the conversion of methane to methyl trifluoroacetate, which include catalysts containing copper[8], manganese[9], and cobalt[10]. As a preliminary step, a variety of transition metals on solid supports were synthesized and screened for activity in a batch reactor system. Details regarding catalyst synthesis and ester production that resulted from this initial screening are given in Table S1. Based on the initial screening, cobalt-containing silica catalysts synthesized via liquid-phase deposition (Co/SiO_2-LPD) and incipient wetness impregnation (Co/SiO_2-IWI) with an aqueous cobalt nitrate solution showed the most promising activity with an absence of significant metal leaching. These materials were therefore selected for further study.

Table S1. Catalytic activity screening of various transition metal-based catalysts

Catalyst	Transition Metal	Support material	Catalyst Mass [mg]	Temperature [°C]	Ester Production [µmol]
Al_2O_3	-	-		200	< 3
Cu/Al_2O_3-LPD	Cu	Al_2O_3	100	200	< 3
Co/Al_2O_3-LPD	Co	Al_2O_3	100	200	4
Co/Al_2O_3-LPD	Co	Al_2O_3	100	215	11
Mn/Al_2O_3-LPD	Mn	Al_2O_3	100	200	< 3
Co/TiO_2-LPD	Co	TiO_2 (rutile)	100	200	< 3
CeO_2	-	-	100	200	< 3
Cu/CeO_2-LPD	Cu	CeO_2	100	200	5
Cu/CeO_2 - IWI	Cu (5 wt%)	CeO_2	100	215	30[a]
Co/CeO_2-LPD	Co	CeO_2	100	200	7
SiO_2	-	-	100	215	5
Co/SiO_2-LPD	Co	SiO_2	100	200	15
Co/SiO_2-LPD	Co	SiO_2	100	215	30
Co/SiO_2-IWI	Co (5 wt%)	SiO_2	100	215	76
Cu/SiO_2-LPD	Cu	SiO_2	100	215	19

Reaction Conditions: 5 bar CH_4, 2 bar air, 7.0 g of 14 wt% TFA/C_6F_{14}, 3 h

[a] Significant leaching detected via hot filtration and visible inspection

Co/SiO$_2$-LPD Catalytic Activity and Characterization

The performance of the Co/SiO$_2$-LPD material was first assessed for the aerobic conversion of methane to methyl trifluoroacetate outlined in Figure 1 of the main text. At 180 ºC, Co/SiO$_2$-LPD shows minimal ester productivity (< 5 μmol/g$_{cat}$.h) in a batch reactor vessel charged with the Co/SiO$_2$-LPD catalyst, 7 g of a 14 wt% TFA in C_6F_{14} solution, 5 bar methane, and 2 bar air. Upon increasing the temperature to 200 ºC, a substantial increase in ester productivity (49 μmol/g$_{cat}$.h) is observed (Figure S3a). The catalytic nature of the Co/SiO$_2$-LPD system for aerobic methane oxidation is confirmed through a series of additional tests listed in Table S2. The use of cobalt-free silica results in negligible productivity, thereby proving the necessity of the transition metal for the oxidation of methane in this process. Likewise, the use of cobalt-loaded silica under otherwise identical reaction conditions in the absence of air results in insignificant ester production, demonstrating that cobalt on silica does not stoichiometrically oxidize methane under the employed reaction conditions.

Upon increasing the reaction temperature to 215 ºC, the Co/SiO$_2$-LPD ester productivity doubles from 49 to 106 μmol/g$_{cat}$.h (Figure S3a). Based on this trend, it is likely that this ester productivity could be further improved through a further increase in temperature. Under the aforementioned reaction conditions, in which methane is in high excess and oxygen is the limiting reactant, the resulting ester yield obtained with 100 mg of the Co/SiO$_2$-LPD catalyst with a 3 h reaction time corresponds to an oxygen conversion to the methyl ester of approximately 5%.

The blue curve in Figure S3b shows the temporal evolution of the ester product during the aerobic oxidation of methane with Co/SiO$_2$-LPD. The heterogeneity of the reaction is confirmed by a typical hot filtration test (See Methods). There is no increase in productivity after the filtration and removal of the solid from the reaction medium (dotted red curve, Figure S3b), indicating the catalysis is genuinely of a heterogeneous nature. Furthermore, ICP-AES measurements on the reaction filtrate did not detect leached cobalt in the solution that could be catalysing the reaction homogeneously.

Characterization of the Co/SiO$_2$-LPD catalyst indicates that the cobalt is highly dispersed in the material. ICP-AES measured the cobalt loading on the Co/SiO$_2$-LPD material to be approximately 0.1 wt%. TEM analysis of the material reveals the absence of distinct cobalt particles and suggests a high dispersion of the cobalt on the silica support (Figure S3c). Furthermore, XRD shows that the cobalt-containing material is comparable to that of the parent silica (Figure S3d), in that no Bragg reflections indicative of the presence of any extended cobalt-containing phases are observable.

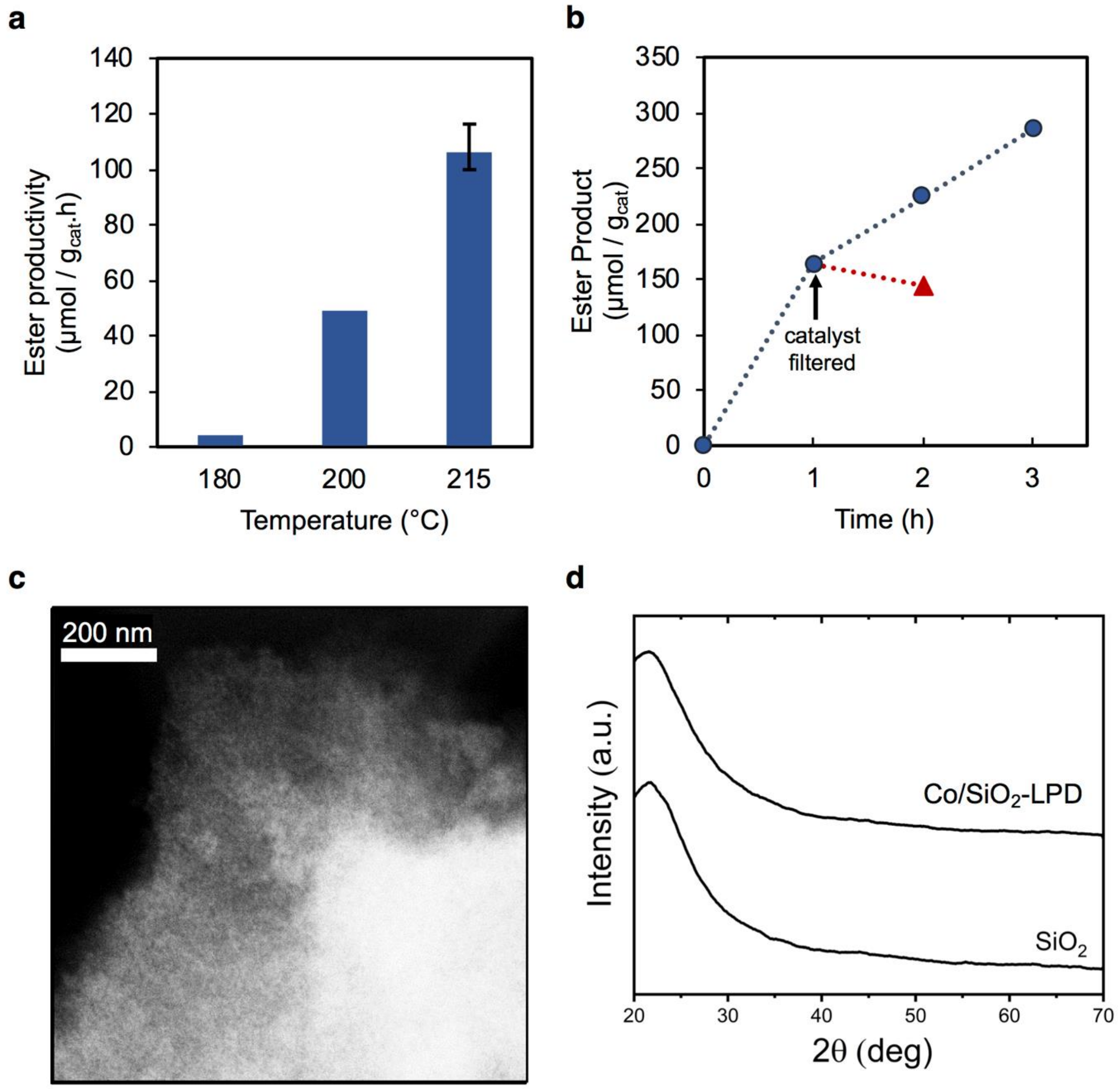

Fig. S3. Co/SiO$_2$-LPD catalytic methane oxidation performance and characterization
(a) Effect of temperature on methyl ester productivity in methane-oxidation. Reaction conditions: 5 bar CH_4, 2 bar air, 7 g of 14 wt% TFA/C_6F_{14}, 100 mg catalyst, 3 h. Data for 215 ºC condition repeated in three trials, average value reported and error bars represent maximum and minimum values (within ±10% from average value); (b) Temporal evolution of methyl ester product during oxidation of methane (blue) and hot filtration test (red) with Co/SiO$_2$-LPD catalyst filtered off after 1 hour. Reaction conditions: same as mentioned above with 100 mg catalyst at 215 °C; (c) Transmission electron micrograph representative of Co/SiO$_2$-LPD catalyst with scale bar of 200 nm; (d) XRD spectra of the silica support material and Co/SiO$_2$-LPD catalyst.

Table S2. Catalytic and aerobic conversion of methane to methyl ester for Co/SiO$_2$-LPD catalyst

Entry	Catalyst	TFA in Fluorous Reaction Medium	CH_4 [bar]	Air [bar]	Ester Yield [µmol]
1	-	14 wt%	5	2	< 3
2	SiO_2	14 wt%	5	2	< 3
3	Co/SiO$_2$-LPD	14 wt%	5	0	< 3[a]
4	Co/SiO$_2$-LPD	14 wt%	5	2	32[b]

Reaction Conditions: 215 ºC, 7.0 g TFA/C_6F_{14} solution, 100 mg catalyst, 3 h
[a] Reaction Conditions: 200 ºC, 7.0 g TFA/C_6F_{14} solution, 100 mg catalyst, 2.3 h
[b] Average ester yield for three repeated trials

Co/SiO2-IWI Catalytic Activity

Figure S4 displays the temporal evolution of the ester product for the 0.5 wt% Co/SiO_2-IWI catalyst, which again shows that the reaction terminates after the filtration and removal of the solid catalyst. Leached cobalt was not detected by ICP-AES in the reaction medium. As such, the activity in the Co/SiO_2-IWI systems can be attributed to heterogeneous catalytic events occurring within the system. The aerobic nature and necessity of cobalt to catalyse the partial oxidation of methane for the Co/SiO_2-IWI system is confirmed in Table S3. Methane- and oxygen-based yields of the methyl ester under various reaction conditions are reported in Table S4 and Table S5, respectively.

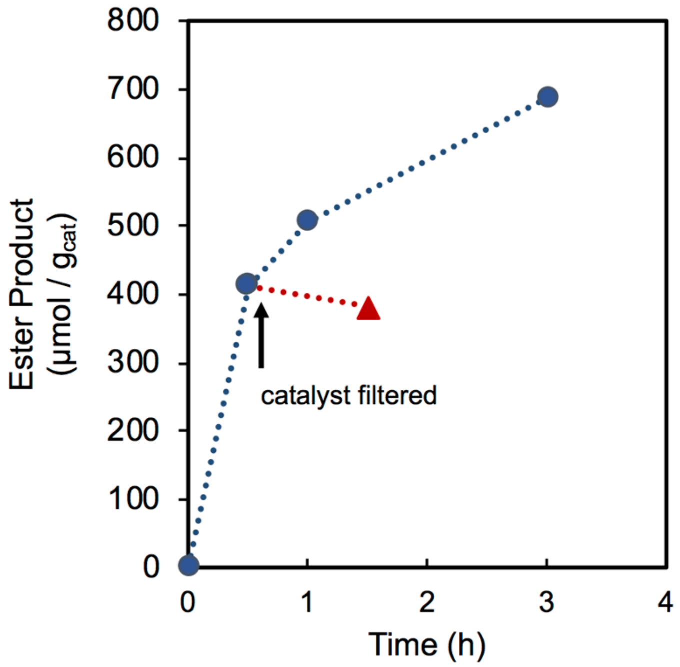

Fig. S4. Hot filtration test for Co/SiO2-IWI
Temporal evolution of methyl ester product during oxidation of methane (blue) and hot filtration test (red) with 0.5 wt% Co/SiO_2-IWI catalyst filtered off after 0.5 h. Reaction conditions: 100 mg catalyst, 5 bar CH_4, 2 bar air, 7 g of 14 wt% TFA/C_6F_{14}, 215°C, 3 h.

Table S3. Catalytic and aerobic conversion of methane to methyl trifluoroacetate for Co/SiO2-IWI catalysts

Entry	Catalyst	TFA in Fluorous Reaction Medium	CH_4 [bar]	Air [bar]	Ester Production [µmol]
1	SiO_2	14 wt%	5	2	< 3
2	CoS-IWI 1.5 wt%	0 wt%	5	2	< 5
3	CoS-IWI 1.5 wt%	14 wt%	0	5	< 5
4	CoS-IWI 1.5 wt%	14 wt%	5	0	< 5
5	CoS-IWI 1.5 wt%	14 wt%	5	2	73

Reaction Conditions: 215 ºC, 7.0 g TFA/C_6F_{14} solution, 100 mg catalyst, 1 h

Table S4. Methane-based yields for Co/SiO$_2$-IWI 1.5 wt% in oxygen-rich conditions

Entry	5% CH_4 in He [bar]	Air [bar]	TFA Conc.	Reaction Time [h]	Ester Production [µmol]	% Yield
1	3	3	14 wt%	1	13	10%
2	3	3	25 wt%	1	21	16%
3	2	4	14 wt%	3	12	14%
4	3	3	14 wt%	5	23[a]	17%[a]

Reaction Conditions: 100 mg 1.5 wt% Co/SiO$_2$-IWI catalyst, 215 ºC, 7.0 g TFA/perfluoroalkane solution
[a] Average value of three repeated trials

Table S5. Oxygen-based yields for Co/SiO$_2$-IWI 1.5 wt% in methane-rich conditions

Entry	CH_4 [bar]	Air [bar]	TFA Conc.	Reaction Time [h]	Reaction Medium [g]	Ester Production [µmol]	% Yield
1	5	2	25 wt%	1	8	53	8%
2	5	2	25 wt%	3	24	35	10%
3	5	1	25 wt%	3	24	23	11%
4	5	1	14 wt%	1	14	19	5%
5	5	1	25 wt%	1	8	41	12 %

Reaction Conditions: 100 mg 1.5 wt% Co/SiO$_2$-IWI catalyst, 215 ºC

Co/SiO_2-IWI Characterization of Catalysts

TEM Images and Particle Size Distribution

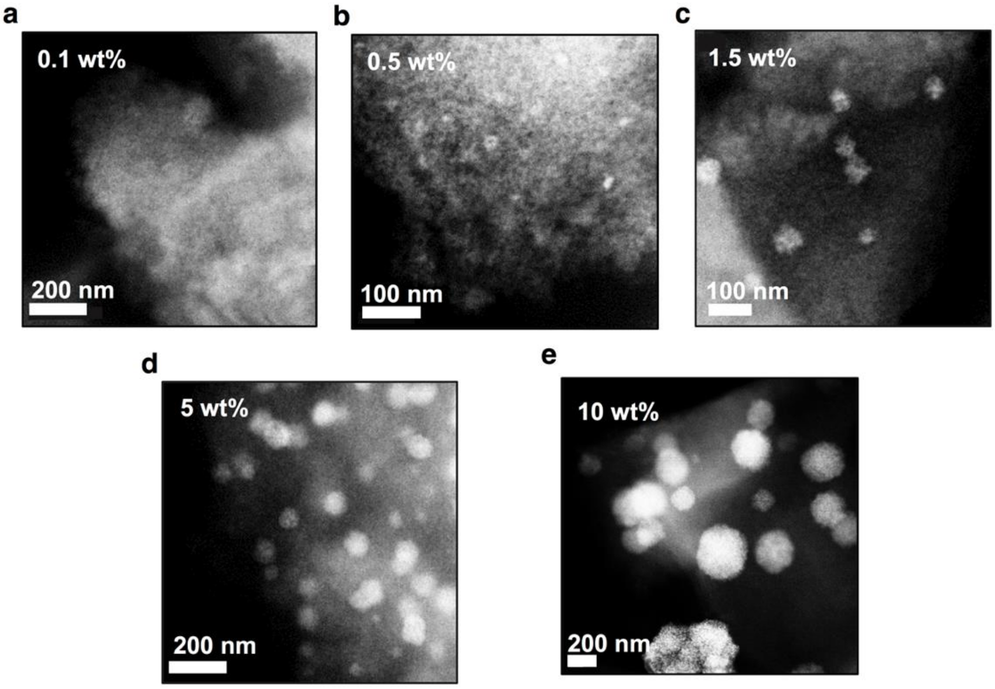

Fig. S5. TEM images of Co/SiO_2-IWI catalysts
(a) TEM image of 0.1 wt% Co/SiO_2-IWI catalyst with scale bar of 200 nm; (b) TEM image of 0.5 wt% Co/SiO_2-IWI catalyst with scale bar of 100 nm; (c) TEM image of 1.5 wt% Co/SiO_2-IWI catalyst with scale bar of 100 nm; (d) TEM image of 5 wt% Co/SiO_2-IWI catalyst with scale bar of 200 nm; (e) TEM image of 10 wt% Co/SiO_2-IWI catalyst with scale bar of 200 nm.

Table S6. Particle size distribution of Co/SiO_2-IWI catalysts based on TEM images

Co Loading	No. Particles	$d_{avgerage}$ [nm]	d_{median} [nm]	St. Dev. [nm]
0.5 wt%	57	22	20	8
1.5 wt%	49	68	64	43
5 wt%	268	100	93	50
10 wt%	77	307	237	251

Note: An insufficient number of cobalt particles on the 0.1 wt% Co/SiO_2-IWI catalyst were identified for this analysis

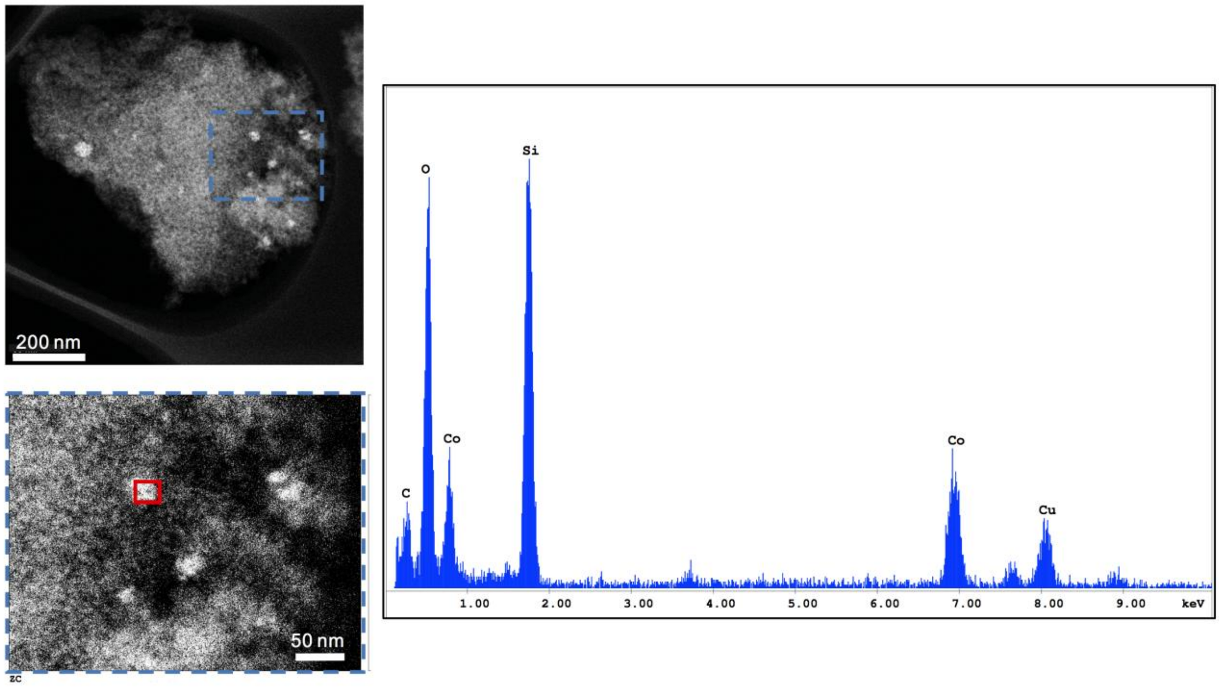

Fig. S6. EDXS representative of Co/SiO_2-IWI catalyst with 0.5 wt% Co loading
The red box in the bottom left image represents the region corresponding to the EDX spectrum presented on the right. The EDXS signals corresponding to Cu and C are a result of sample preparation.

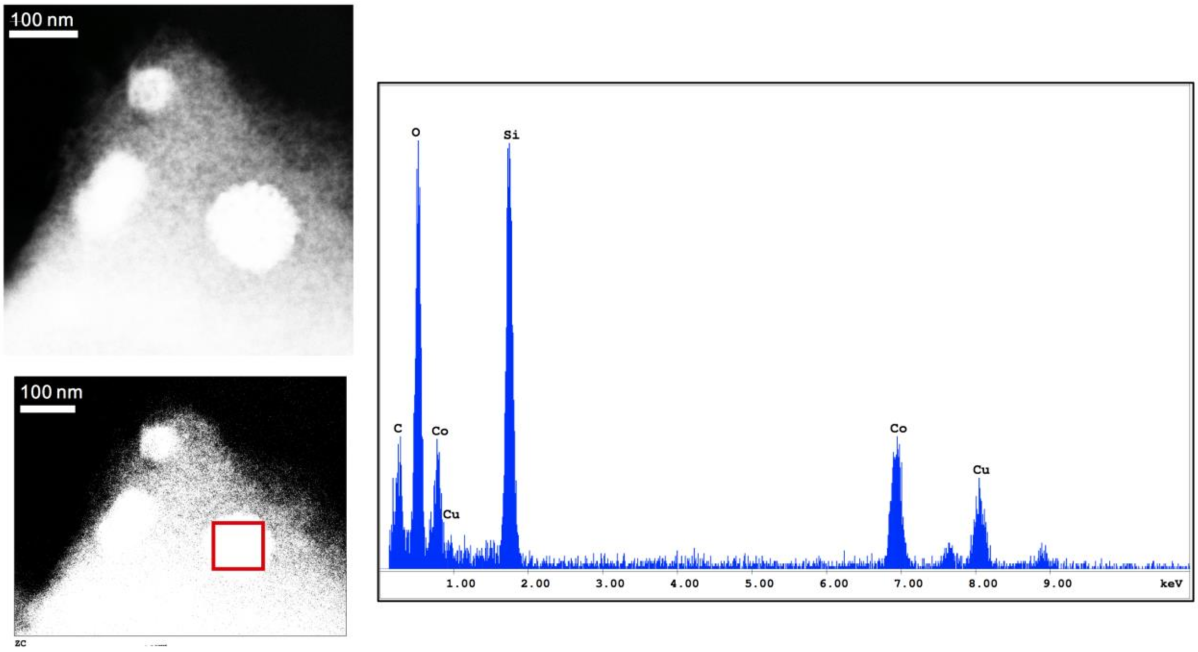

Fig. S7. EDXS representative of Co/SiO_2-IWI catalyst with 1.5 wt% Co loading
The red box in the bottom left image represents the region corresponding to the EDX spectrum presented on the right. The EDXS signals corresponding to Cu and C are a result of sample preparation.

XRD

The presence of Co_3O_4 particles is apparent through the reflection at a 2θ value at approximately 37° (Figure S7). The reflection assigned to the (311) plane of Co_3O_4 starts to appear as a minor bump in the XRD diffractogram of the 0.5 wt% catalyst, and then grows into a prominent reflection for loadings exceeding 1.5 wt%. For the 5 wt% and 10 wt% catalysts, several reflections corresponding to other (hkl) planes are also observed[11].

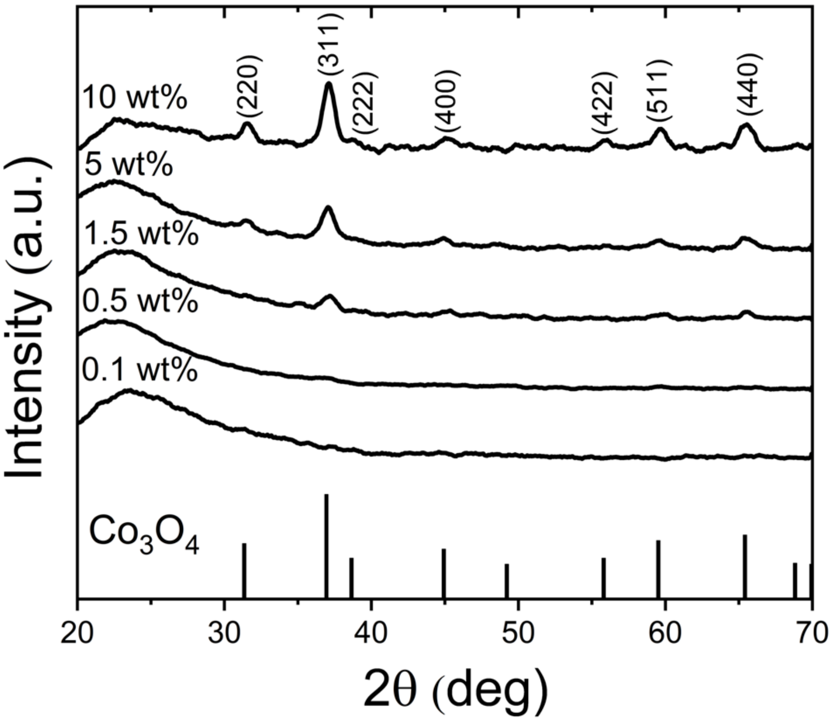

Fig. S8. XRD of Co/SiO$_2$-IWI catalysts in fresh state
Reflections labeled based on a Co_3O_4 reference (Ref No: 98-001-0247).

Co K-Edge XANES in Fresh State

Across the range of weight loadings, small variations are observable in the Co K-edge XANES of the fresh catalysts (Figure S9). Notably, in the case of the 0.1 wt% Co/SiO_2-IWI catalyst, there is a small shift to a lower binding energy and change in the shoulder feature. A two-component linear combination analysis (LCA) for the Co/SiO_2-IWI catalysts using the bulk Co_3O_4 and $Co(OH)_2$ standards indicates the dominance of a Co_3O_4-type cobalt species with a much smaller fraction of a second cobalt phase which has some resemblance to a $Co(OH)_2$-like species (Figure S9 inset,). The lowest-loaded sample contains the largest fraction of this $Co(OH)_2$-like phase. The corresponding EXAFS (Figure S12) additionally confirms the spinel Co_3O_4 structure as the principal cobalt phase in the fresh catalysts.

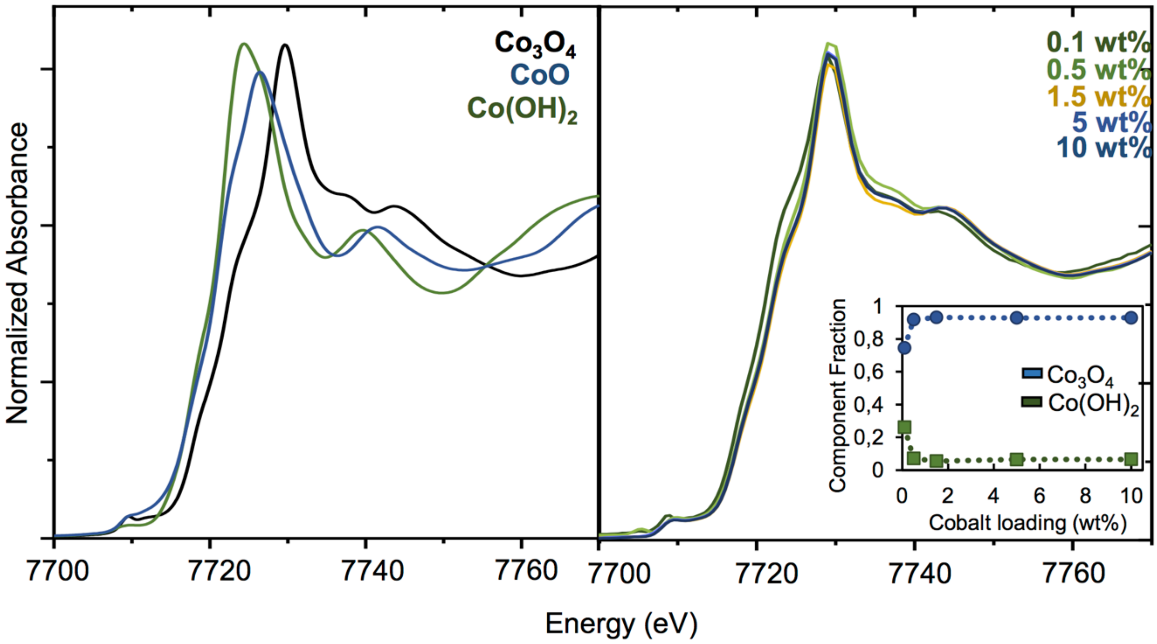

Fig. S9. Co K-edge XANES of Co_3O_4, CoO, and $Co(OH)_2$ standards and Co/SiO_2-IWI catalysts in fresh state

Inset includes the results of a two-component LCA of the 0.1 wt% - 10 wt% Co/SiO_2-IWI samples with the Co_3O_4 and $Co(OH)_2$ standards.

LCA Analysis of the Co K-Edge XANES

Figure S10 gives an overview of the LCA fitting of fresh, spent, and regenerated Co/SiO_2-IWI catalysts over the 0.1 wt% ≤ [Co] ≤ 10 wt% range of cobalt loadings from the perspective of the statistical goodness of fit (X^2) achieved by different approaches to the LCA founded upon bulk references for Co_3O_4, CoO, and/or $Co(OH)_2$. For the Co/SiO_2-IWI catalysts in their fresh state, the spectra appear to be equally well described by a combination of only two components, although it cannot be specified whether the second component is more of a CoO or $Co(OH)_2$ nature on this basis. Statistically, a three-component approach is not justified, as the statistical improvement to the fits achieved through adding the third component is not significant. For the Co/SiO_2-IWI catalysts in spent and reactivated states, the X^2 values increase significantly as the cobalt weight loading decreases, and there is a clear preference for the two-component LCA fit with the bulk Co_3O_4 and $Co(OH)_2$ standards at loadings below 1.5 wt%. The absolute values of X^2 for ≤ 0.5 wt% Co/SiO_2-IWI spent and reactivated samples are almost an order magnitude greater than the X^2 for the corresponding fresh samples. The results of these fits suggest that: i) post-catalysis, the ≤ 0.5 wt% Co/SiO_2-IWI catalysts may no longer well-described by the bulk standards; and ii) the character of the Co^{II} component appears to have more $Co(OH)_2$ character than CoO.

Figure S11 gives two examples (0.1 wt% and 5 wt%, as indicated) of the results of the LCA fitting of the XANES together with the residuals which result. For the 5 wt% Co/SiO_2-IWI catalyst, the spent catalyst shows enhanced levels of residuals to the fit in the region where a pronounced Co^{2+}-like shoulder is seen to develop at ca. 7725 eV in the spent sample. Upon reactivation, these residuals are decreased, with the nature and magnitude of the remaining residuals closely resembling those of the fresh catalyst, indicating a restoration of the starting nature of the material. For the 0.1 wt% Co/SiO_2-IWI catalyst, more substantial changes are observed after catalysis that are maintained even after application of the aerobic reactivation protocol. The XANES envelope in this dilute case is characterized by a very strong white line at ca. 7725 eV, and is significantly different in global terms compared to all the other cases. As a result the ability of the two standards employed to describe this region is severely diminished.

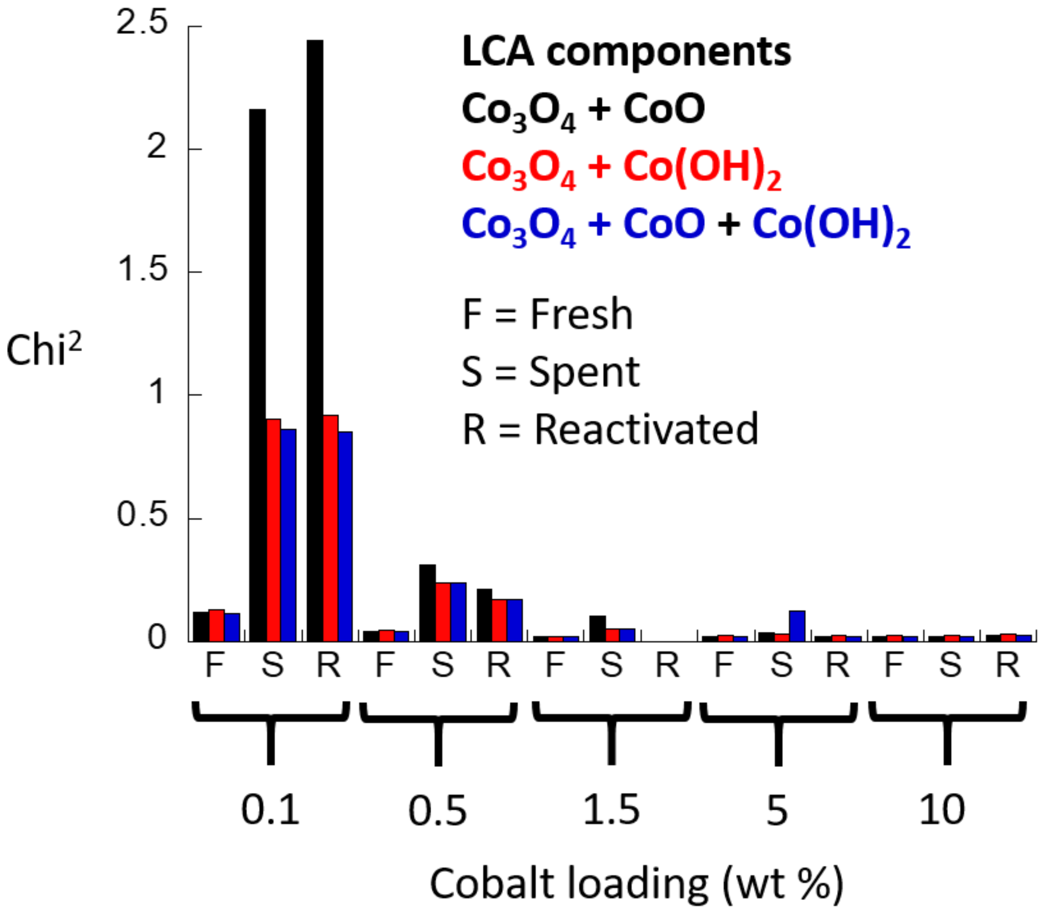

Fig. S10. Variation of X^2 resulting from three different approaches to a LCA fitting of the Co K-edge XANES for the Co/SiO_2-IWI catalysts in their fresh, spent, and reactivated states

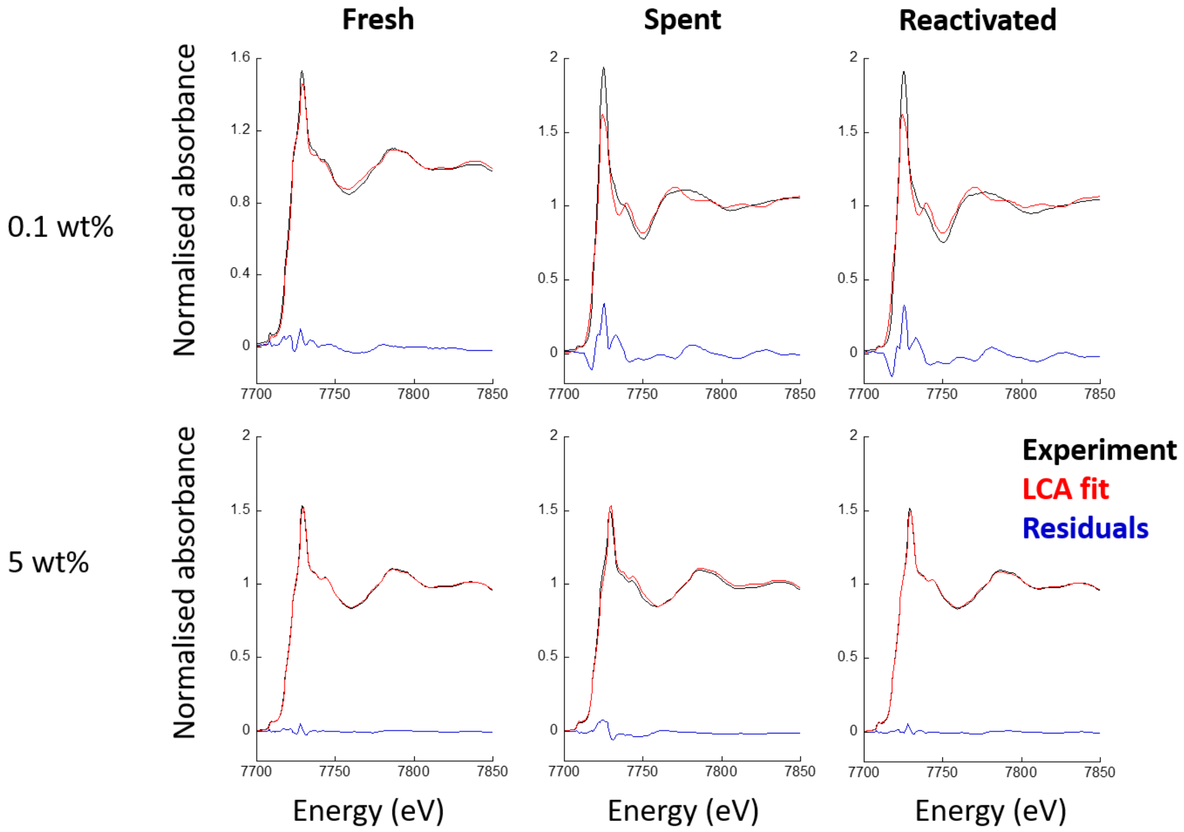

Fig. S11. Comparison of the fits to Co K-edge XANES achieved from the two-component LCA (Co_3O_4 + $Co(OH)_2$) for the 0.1 wt% and 5 wt% Co/SiO_2-IWI catalysts in the fresh, spent, and regenerated states

Co K-Edge EXAFS in Fresh State

The left hand panel of Figure S11 shows the k^3-weighted EXAFS obtained from the 0.1 wt% ≤ [Co] ≤ 10 wt% series of Co/SiO_2-IWI catalysts together with (black curve) that derived from a Co_3O_4 bulk standard. The right hand panel of Figure S12 then gives the corresponding non-phase corrected Fourier transforms of the k^3-weighted data in each case, along with that of the standard.

Across the loading range, all fresh Co/SiO_2-IWI catalysts show a strong spinel Co_3O_4 structural motif. At lower weight loadings, the only significant changes in the EXAFS envelope are reductions in the intensity of the Co-Co scattering interactions pertaining to the first nearest neighbors in the octahedrally coordinated (2.87 Å, Co^{3+}), and tetrahedrally coordinated (3.36 Å, Co^{2+}) centers present within the spinel structure. The first (Co – Co) scattering interaction that exists between these two different Co sites (Co_{Oct} - Co_{Td}, ca. 5 Å) also attenuates over this range of loadings, and across the range of diminishing domain sizes indicated by electron microscopy. Nonetheless, the Co_3O_4 structure predominates across the range of samples studied.

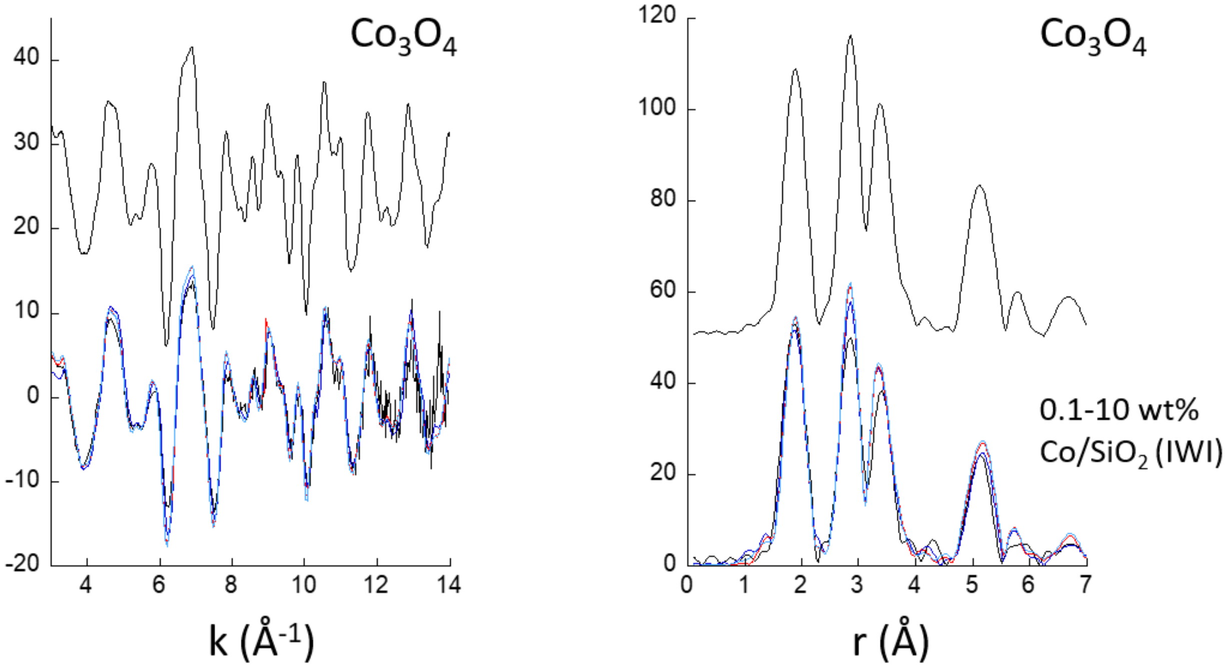

Fig. S12. EXAFS of all Co/SiO_2-IWI catalysts in fresh state
(a) k^3-weighted EXAFS and (b) phase corrected Fourier transform representation of the k^3-weighted EXAFS for the series of fresh Co/SiO_2-IWI catalysts over the cobalt loading range of 0.1 wt% (black) to 10 wt% (light blue). The EXAFS from a bulk Co_3O_4 reference sample is also given for comparison.

Co K-Edge XANES and EXAFS in Spent State

Figure S13 gives (left hand panel), normalized Co K-edge XANES derived from the spent catalysts across the 0.1 wt% ≤ [Co] ≤ 10 wt% range of loadings along with the XANES spectra due to the standards employed for LCA analysis. The right hand panel of Figure S13 then gives the Fourier transforms of the k^3-weighted EXAFS for the same range of spent samples and standards, as indicated. It can be observed that, post-reaction, the low binding energy feature (ca. 7725 eV) to the Co K-edge XANES develops and becomes more prominent as the loading of cobalt is decreased. The exception to this is found at 0.1 wt% sample, where what was previously a shoulder develops into the dominant feature within the XANES of this catalysts after catalytic reaction.

For cobalt loadings between 0.5 wt% and 10 wt%, the EXAFS (right hand panel of Figure S13) shows that the predominant Co_3O_4 structural motif, which was found to dominate the habit of the cobalt across this loading range, is preserved. However, the 0.1 wt% Co/SiO_2-IWI deviates significantly from this trend. In this case, the EXAFS shows that the Co_3O_4 structure has been replaced, post-catalysis, and the formation of a new, highly dispersed, and/or disordered cobalt structure is indicated. In combination with results from the reactivated sample shown in Figure S11, this new structure for the 0.1 wt% Co/SiO_2-IWI is shown to be retained after the reactivation step, whereas the higher weight-loaded catalysts show a partial or complete restoration of the starting cobalt phase.

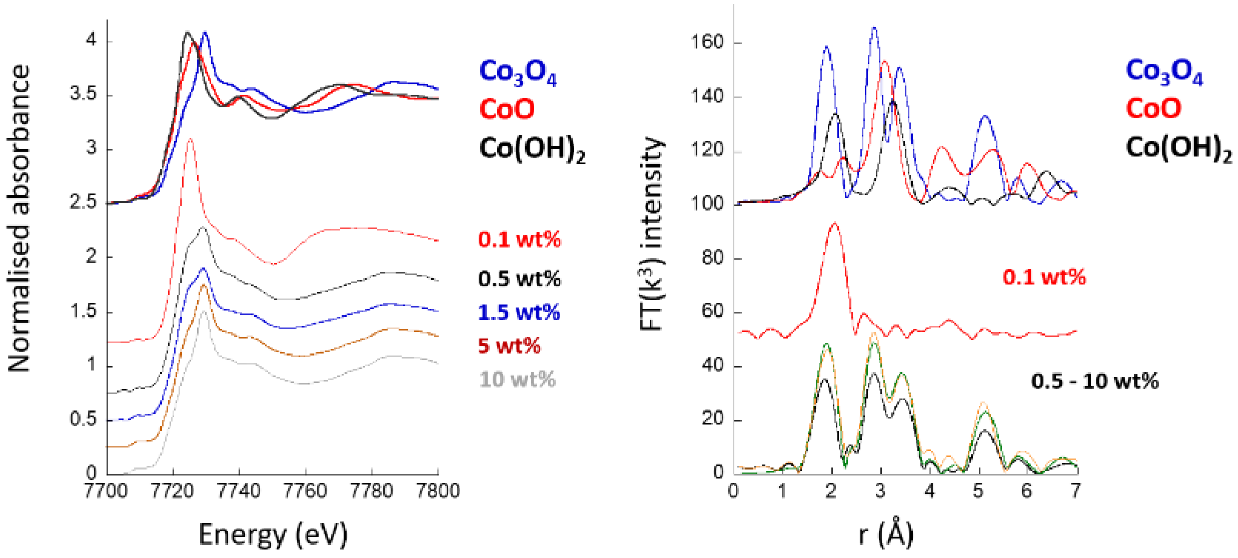

Fig. S13. XAS summary of all Co/SiO_2-IWI catalysts in spent state
(a) Co K-edge XANES of spent Co/SiO_2-IWI catalysts and the three standards; (b) Corresponding Fourier transform representations of the k^3-weighted Co K-edge EXAFS for each sample and standard.

EXAFS Fitting of the Spent 0.1 wt% Co/SiO₂-IWI Catalyst

In the 0.5 ≤ [Co] ≤ 10 wt% range of cobalt loadings, simple inspection of the Co K-edge EXAFS shows that, in the spent and regenerated cases, the Co_3O_4 spinel structure is retained throughout. However, as indicated by the Co K-edge XANES shown in the main paper and above a far more radical change in cobalt speciation results from the application of reaction conditions in the 0.1 wt% Co/SiO_2-IWI case. Moreover, it is also evident from the Co K-edge XANES that this structural change is not reversed by the aerobic reactivation protocols subsequently applied. However, it is also evident from the Co K-edge XANES that this structural change is not reversed by the aerobic reactivation protocols subsequently applied, yet the system retains activity (Figure 2 (e), main paper).

The XANES alone (Figure S9) indicate a complete replacement of the Co_3O_4 spinel phase initially present. In its place, a new Co^{II} phase, of significantly different symmetry (from changes in the XANES pre-edge region) has been created by the catalysis. The FT of the k^3-weighted EXAFS from the spent 0.1 wt% Co/SiO_2-IWI case (Figure S13), further suggests that the cobalt in this new phase is very highly dispersed. In this case, therefore, EXAFS analysis has been used in an attempt to establish more information regarding the nature of this new phase.

Figure S14 reports fittings of the EXAFS based upon two candidate symmetries, six-coordinate O_h and four-coordinate T_d cobalt, and using a full curved wave multiple scattering approach to the analysis of the EXAFS. From this initial fitting it can be seen that the six-coordinate O_h motif is clearly favored over the four-coordinate T_d case, both in terms of how well the first (Co-O) shell us described and how and the contributions of multiple scattering manifest.

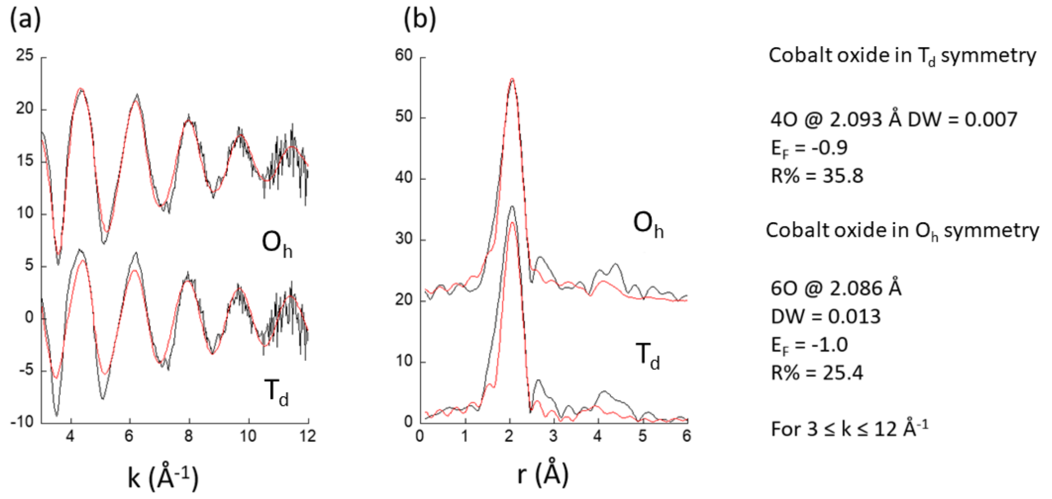

Fig. S14. Initial fitting of the Co K-edge EXAFS derived from the 0.1 wt% Co/SiO_2-IWI sample using full cured wave multiple scattering theory for two candidate symmetries as indicated.
(a) k^3-weighted EXAFS, and (b) the Fourier transform representation of the k^3-weighted EXAFS. In each case, the black lines refer to the experimental data and the red lines to the theoretical fits to that data.

Figure S15 shows the k^3-weighted EXAFS and the corresponding phase-corrected Fourier transforms of the k^3-weighted EXAFS for the 0.1wt% Co/SiO_2-IWI catalyst, along with a number of example fits to this data (in red) achieved using six models (as indicated) based on single scattering (SS) or multiple scattering (MS) approaches to analysis. In the latter case, the overall symmetry of the model (O_h or d_2h is also given). Table S7 then reports the structural and statistical parameters derived from these models.

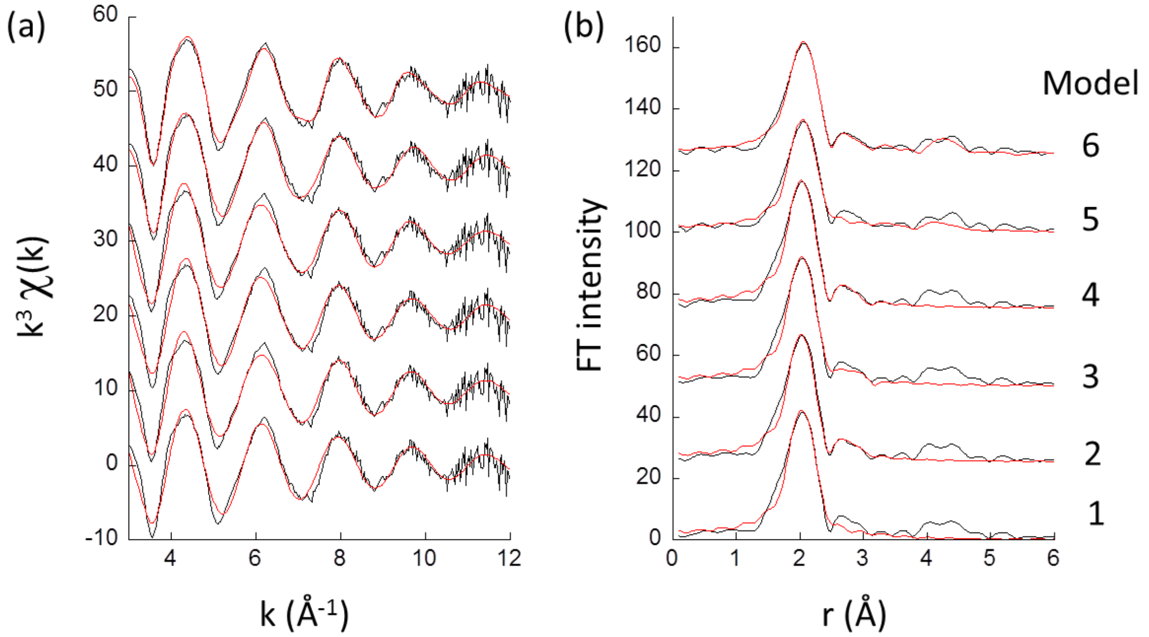

Fig. S15. EXAFS analysis for 0.1 wt% Co/SiO_2-IWI in spent state
(a) k^3-weighted EXAFS derived from the spent 0.1 wt% Co/SiO_2-IWI catalyst, and (b) the corresponding phase-corrected Fourier transforms, for six different models of the EXAFS. Models 1 to 4 are based upon single scattering, (SS) plane wave, theory whilst models five and six utilize full curved wave multiple scattering (MS) theory for a maximum of three contributing atoms. In each case, the black curves are the experimental data, the red, the fits due to the analyses made using EXCURV*[12]*.

From Figures S14 and S15, the majority of the EXAFS (k^3) scattering envelope can be very well described via a first shell comprising of 6 oxygen atoms at a distance of 2.09 Å. Subsequent to this, a further shell, comprised of either Co-Si or Co-Co interaction at a distance of ca. 2.68 or 2.89 Å respectively, can also be fitted to this data. Modelling this second shell with a mixed occupancy (overlapping contributions from Co-Co scattering and Co-Si scattering) is also found to be possible, although the change in goodness of fit achieved through the addition of these shells is marginal.

Indeed, as can be seen from the addition of a model (shown in Figure S14 and again as (5) in Figure S15) based solely around a six-coordinate O_h cobalt species, the biggest impact on the fitting of the data can be seen to arise from the inclusion of multiple scattering effects due to an undistorted octahedral symmetry. Tests (to include MS effects) were also made (not shown) of a square based pyramidal (SBP) arrangement, wherein the total Co-O coordination is 5 (split into a 4 + 1 arrangement to yield a C_4

symmetry), and a single Si linkage using to complete the overall octahedral environment suggested by the above. However, refinements based on this model result in the DW factor associated with the single atom occupancy in one of the oxygen shells being negative. This physically unreasonable result constitutes further evidence that the average Co-O coordination is 6 and not 5.

Given that Co^{II} has a d^7 electron count within a O_h ligand field with unpaired electrons in both e_g (dz^2 or $d(x-y)^2$) orbitals and t_2g (d_{xy}, d_{yz}, d_{xz}) orbitals, and that the selective activation of methane required two electrons to be handled, then we might further postulate that the O_h Co^{II} centers may in fact be $Co(O)_5(OH)$ and/or $Co(O)_5(OCH_3)$.

The evidence from the XAS suggests that the required electrons are handled via a reduction of Co^{III} (present in the starting Co_3O_4 phase) to Co^{II} and, therefore, that two Co centers are required to be involved. The above stated speciation would then lead to the formation of two 18 e^- complexes with the unpaired electron in the dz^2 orbital pairing with the electron supplied by the ligand (be it OH or CH_3O^-), which may be considered as potentially stable monomers.

However, there remains the further possibility: that these species, which arise from a common and localized nanoparticulate Co_3O_4 source, could retain further interactions between these monomeric species that may further lower their energy through pairing electrons in the $d(x-y)^2$ orbitals to yield dimeric or oligomeric species. In such a case, two octahedra may come together through either sharing edges, or through sharing corners, as is found within the cubic structure of $Co^{II}O$ and other systems (see for example [S1]). In $Co^{II}O$ edge-sharing octahedral appear with a Co-Co distance of 3.03Å, whereas the corner sharing Co-Co distance is significantly longer (4.29 Å), though in other systems these distances can be significantly shorter[13].

As such, and in spite of the marginal influence of the second shell (be it comprised of Si (model 2), Co (model 3), or a mix thereof, (model 4)), when fitted using an SS approach, we also report the results of a further model. This model aims to test a least one of the above-mentioned possibilities, namely, that of edge-sharing Co^{II} octahedra using full curved wave MS (model 6). This is found to yield the best description of the experimental EXAFS. It is the case, however, that in terms of the number of parameters used to obtain a certain degree of fit to the k^3-weighted EXAFS, one would prefer the fit based solely on that derived from the putative monomer, O_h Co^{II}, when MS is taken into account.

The results of model 6, however suggest that the Co speciation present could equally be comprised, at least in part, of edge-sharing Co^{II} octahedra; the derived Co-Co bond distance being compatible with this notion. However, from this model, and in respect of the symmetry applied, the coordination for this shell (2) is fixed by the symmetry and cannot be refined; and a coordination number of two for this shell

would suggest an average nuclearity or at least 3 (a symmetric trimer or a linear array) or above. However, if we further take into account model 3, which permits the coordination to be refined, but which does not permit the contribution to the higher (4 -5 Å) to be taken into account, we find that a Co-Co coordination of 0.8 arises. This would suggest, though at this point it does not constitute a proof, this system may at least in part be comprised of edge sharing Co^{II} dimers

As to the possibility of the presence of corner sharing Co^{II} octahedra. Whilst the higher shell structure, which contains within it contributions from MS resulting from the O_h environment, exists precisely in the distance range where we might expect corner-sharing Co^{II} octahedra to have their principal Co-Co scattering interactions, attempts to model this and confirm whether these interactions exist or not have proved inconclusive. The most likely reasons for this are deemed to be: the finite length and statistical quality of the data, which limits the number of parameters that may be reasonably be refined; the likely complexity of contribution that could be contributing at these distances from the central Co atom, and; the limitations associated with how reasonably model such hypothetical structures. As such, whilst a two cluster approach, where a partial occupancy can be attributed to both edge-sharing and corner-sharing species and full curved wave MS theory applied, can be used to fit the data, it remains unclear as to just how reliable such a fitting is for the corner sharing component. Therefore, at the present time, we consider that only the presence of monomeric or edge-sharing dimeric species (models 4 and 6) can realistically be supported on the basis of the data obtained.

Table S7. Structural and statistical data arising from analysis of Co K-edge EXAFS from the spent 0.1 wt% Co/SiO_2-IWI catalyst for the six models shown in Figure S14

K_{min} = 3, k_{max} = 12, AFAC = 0.9

Model	Elements	SS/MS/sym.	CN[a]	R(Å)[b]	DW[c] (2σ2 (Å²))	R(%)[d]	E_F (eV)[e]	M[f]
1	O	SS	6	2.09	0.013	29.4	0.25	4
2	O	SS	6	2.09	0.013	28.0	-0.18	7
	Si		1.4	2.68	0.030			
3	O	SS	6	2.09	0.014	28.8	0.34	7
	Co		0.8	2.89	0.030			
4	O	SS	6	2.09	0.014	27.6	-0.1	10
	Si		1.2	2.70	0.029			
	Co		0.7	2.83	0.036			
5	6O	Oh, MS	6	2.09	0.013	25.3	0.94	4
6	O	d_2h, MS	4	2.12	0.005	22.0	1.2	9
	O		2	2.00	0.004			
	Si		2	2.79	0.031			
	Co		2	2.83	0.033			

AFAC = parameter relating to the fraction of photo-electrons contributing to the EXAFS
SS = single scattering, plane wave
MS = multiple scattering, Curved wave
Sym = applied symmetry
[a] N = Coordination number
[b] R = bond distance
[c] DW = Debye-Waller (disorder) factor ($2\sigma^2$) where σ^2 = mean squared displacement of the atom pair with respect to each other
[d] $R\% = \Sigma_i^N 1/\sigma_i (\chi_i^e(k) - \chi_i^t(k))^2 \times 100\%$ Where χ_i^e and χ_i^t are the experimental and theoretical EXAFS respectively, and k is the photoelectron wave vector (Å^{-1}). σ_i is the uncertainty in the data, with $1/\sigma_i = k_i^n / \Sigma_j^N k_j^n (\chi_i^e(k_j))^2$.
[e] E_F = Fermi energy
[f] From the Nyquist theorem, the number of permisable fitting parameters, where $M = 2\delta K\delta R/\pi + 2 \approx$ 13-14

Fluorous Co-Solvent Selection and Effects

A variety of perfluorinated solvents are commercially available and can, in theory, be interchanged in the system. However, not all perfluorinated solvents are suitable due to their low boiling points, toxicity, or additional hazards. The primary criteria for the selection of a fluorous co-solvent are inertness and stability under the reaction conditions, such that unwanted side-reactions and decomposition products are avoided. Easy handling at room temperature and low toxicity are also requirements. Perfluoroalkanes emerged as strong candidates for the initial development of the system, although other suitable inert fluorous solvents exist and warrant further consideration. While no substantial differences are observed from a catalytic standpoint between reactions with carried out in a perfluorohexane and a perfluorooctane co-solvent for the reported catalysts (Figure S16), selection of the co-solvent could have implications from a process perspective. Differences in the properties of various fluorous co-solvents, such as the solubilities of relevant gases, boiling points, and partitioning of the desired product during the extraction step, provide greater flexibility in developing a more optimized process in the future.

It should also be emphasized that the fluorous character of the co-solvent contributes additional significant advantages in the product separation steps. The pronounced immiscibility of fluorous compounds in non-fluorous solvents results in a very sharp phase separation. After the product is extracted from the fluorous phase, the fluorous solvent can be easily decanted and recycled, reducing the overall usage in the system and therefore the associated costs of using such a material.

Most of the catalytic data presented in the main text was obtained using perfluorohexane as the fluorous co-solvent for TFA. The amount of the methyl ester product obtained with the 1.5 wt% Co/SiO_2-IWI catalyst remains virtually unchanged upon switching the co-solvent from perfluorohexane to perfluorooctane while keeping the rest of the reaction conditions unchanged (Figure S16). Additionally, the TEM images of the 0.5 wt% and 1.5 wt% Co/SiO_2-IWI catalysts after the initial reaction reveal the presence of cobalt particles, similar to those observed with the freshly prepared catalysts (Figure S17). ICP-AES of the reaction filtrates detected no leached cobalt. Since the solubility of gases, such as oxygen, are greater in perfluorooctane than in perfluorohexane[14], the independence of the catalytic activity from the perfluoroalkane co-solvent could suggest that the reaction is primarily kinetically-controlled under the stated reaction conditions.

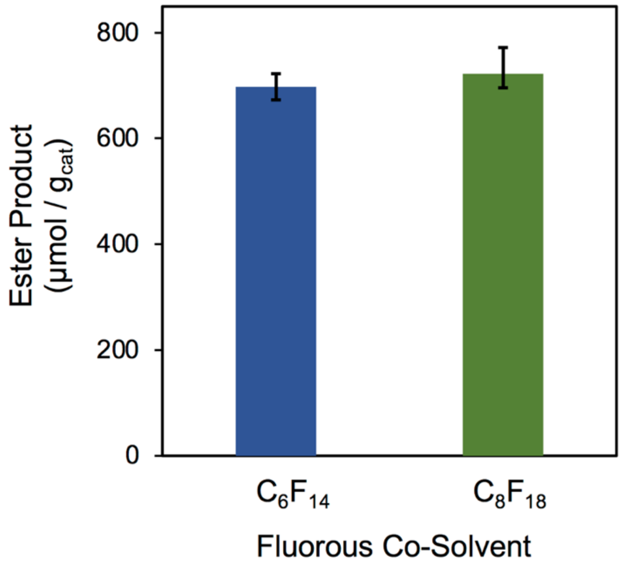

Fig. S16. Effect of nature of perfluorocarbon co-solvent on activity of 1.5 wt% Co/SiO_2-IWI catalyst
Reaction conditions: 5 bar CH_4, 2 bar air, 7 g of 14 wt% TFA/perfluorocarbon, 215 °C, 100 mg catalyst, 1 h. Data for both conditions repeated in 2-3 trials, average value reported and error bars represent maximum and minimum values (within ±10% from average value).

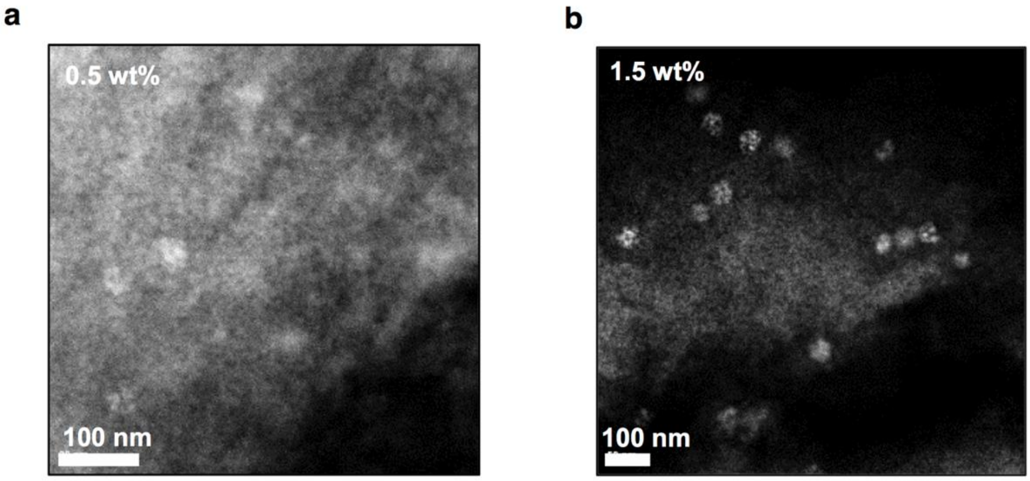

Fig. S17. TEM of Co/SiO_2-IWI catalyst in spent state
a) TEM image with scale bar of 100 nm of 0.5 wt% Co/SiO_2-IWI catalyst after reaction; (b) TEM image with scale bar of 100 nm of 1.5 wt% Co/SiO_2-IWI catalyst after reaction.

Ester Hydrolysis

The product protection strategy employed in this study involves the conversion of methanol in an acid solvent to the corresponding methyl ester. It is possible to hydrolyze the methyl esters into methanol in subsequent steps. The hydrolysis reaction is often regarded as facile and not a primary focus of most work in this area of methane conversion. However, slow reaction kinetics and the re-concentration of the acid could pose significant challenges to the overall process. In general, the separation and hydrolysis of the ester with acid recycle would be influenced by a number of factors including the ester concentration, the kinetics of ester hydrolysis under relevant conditions, and the sensitivity of the catalytic system to water (determining the required degree of acid re-concentration). Although distillation and hydrolysis schemes have been generally proposed[10, 15], none of these proposals are fully interpreted in the context of methane-to-methyl-ester systems under realistic conditions, and we are not aware of any rigorous evaluations of these post-catalysis steps. Considering the potential impact of these steps on a commercial process, we underscore the importance of these evaluations and the development of new solutions for ester hydrolysis and acid recycle in future work.

REFERENCES

[1] O. Muller, M. Nachtegaal, J. Just, D. Lutzenkirchen-Hecht, R. Frahm, *J Synchrotron Radiat* **2016**, *23*, 260-266.
[2] K. V. Klementie, K. V. Klementie www.cells.es/Beamlines/CLAESS/software/xafsmass.html, p. freeware.
[3] A. H. Clark, J. Imbao, R. Frahm, M. Nachtegaal, *J Synchrotron Radiat* **2020**, *27*, 551-557.
[4] A. H. Clark, P. Steiger, B. Bornmann, S. Hitz, R. Frahm, D. Ferri, M. Nachtegaal, *J Synchrotron Radiat* **2020**, *27*, 681-688.
[5] N. Binsted, (Ed.: N. Binsted), CCLRC Daresbury Laboratory Computer Program, **1998**.
[6] S. J. A. Figueroa, C. Prestipino, *J Phys Conf Ser* **2016**, *712*.
[7] aF. Van den Schoor, F. Verplaetsen, J. Berghmans, *Journal of Hazardous Materials* **2008**, *153*, 1301-1307; bS. Kondo, K. Takizawa, A. Takahashi, K. Tokuhashi, *Journal of Hazardous Materials* **2011**, *187*, 585-590; cG. Cui, C. Yang, Z.-l. Li, Z. Zhou, J.-l. Li, *Journal of Loss Prevention in the Process Industries* **2016**, *41*, 252-258; dL. Huang, Y. Wang, S. Pei, G. Cui, L. Zhang, S. Ren, Z. Zhang, N. Wang, *Energy* **2019**, *186*, 115840.
[8] M. Ravi, J. A. van Bokhoven, *Chemcatchem* **2018**, *10*, 2383-2386.
[9] W. S. Chen, J. A. Kocal, T. A. Brandvold, M. L. Bricker, S. R. Bare, R. W. Broach, N. Greenlay, K. Popp, J. T. Walenga, S. S. Yang, J. J. Low, *Catal Today* **2009**, *140*, 157-161.
[10] T. Strassner, S. Ahrens, M. Muehlhofer, D. Munz, A. Zeller, *Eur J Inorg Chem* **2013**, *2013*, 3659-3663.
[11] W. L. Roth, *Journal of Physics and Chemistry of Solids* **1964**, *25*, 1-10.
[12] aS. J. Gurman, N. Binsted, I. Ross, *J Phys C Solid State* **1984**, *17*, 143-151; bS. J. Gurman, N. Binsted, I. Ross, *J Phys C Solid State* **1986**, *19*, 1845-1861.
[13] B. J. Kim, E. Fabbri, D. F. Abbott, X. Cheng, A. H. Clark, M. Nachtegaal, M. Borlaf, I. E. Castelli, T. Graule, T. J. Schmidt, *J Am Chem Soc* **2019**, *141*, 5231-5240.
[14] A. M. A. Dias, M. Freire, J. A. P. Coutinho, I. M. Marrucho, *Fluid Phase Equilibr* **2004**, *222*, 325-330.
[15] J. Yuan, L. Wang, Y. Wang, *Industrial & Engineering Chemistry Research* **2011**, *50*, 6513-6516.
